# Spatial distribution and risk assessment of dengue incidence at district level across major climatic zones in India

**DOI:** 10.1371/journal.pone.0350325

**Published:** 2026-06-09

**Authors:** Meenu Mariya James, Karuppusamy Balasubramani, Praveen Balabaskaran Nina, Natarajan Gopalan, Sujit Kumar Behera

**Affiliations:** 1 Department of Epidemiology and Public Health, Central University of Tamil Nadu, Thiruvarur, Tamil Nadu, India; 2 Department of Geography, School of Earth Sciences, Central University of Tamil Nadu, Thiruvarur, Tamil Nadu, India; 3 Department of Public Health and Community Medicine, Central University of Kerala, Kasaragod, Kerala, India; World Health Organization, Regional Office for South-East Asia, INDIA

## Abstract

Over the past decade, dengue incidence has been steadily increasing across the different climatic zones of India. The role of climatic variability in the spatial and temporal distribution of dengue at the district level across India is to be determined. District-level dengue incidence data from 2010 to 2022 were obtained from the National Centre for Vector-Borne Disease Control. Indian districts were categorized into eleven climatic zones based on the Köppen-Geiger climate classification scheme and subsequently grouped into three significant climatic zones (tropical, temperate and arid). Temporal trends were assessed using the Prais-Winsten regression model that accounted for serial autocorrelation, while climate zonal differences in annual incidence were evaluated using Kruskal-Wallis tests and pairwise comparisons. The global Moran’s I test was used to assess overall spatial autocorrelation, followed by Anselin’s local Moran’s I test to identify clustering and hotspots of dengue incidences at the district level. There is significant heterogeneity in dengue distribution across the districts in India. Prais-Winsten regression analysis shows the strongest upward trend in dengue incidence in polar tundra (ET) zone [AIR = 126.9%; p = 0.01], temperate, no dry season, hot summer (Cfa) zone [AIR: 94.8%; p = 0.01], and cold, no dry season, warm summer (Dfb) zone [AIR = 85.1%; p < 0.001], indicating a substantial intensification of dengue transmission even in cooler climatic regions. Kruskal-Wallis tests confirmed persistent and significant disparities between tropical, temperate, and arid regions. Spatial analysis revealed clustering (Global Moran’s I = 0.06, p < 0.001), with 31 high-incidence clusters concentrated primarily in the semi-arid regions of Punjab and Haryana, and humid regions of Tamil Nadu and Kerala. Overall, the identified clustering of high-incidence districts in semi-arid and humid regions, along with the upward trends in multiple climatic zones, highlights an urgent need to embed climate-sensitive planning, early warning systems, and geographically targeted vector-control measures into India’s dengue prevention framework.

## Introduction

Dengue fever, a neglected tropical disease, is caused by infection with one of four serotypes of the dengue virus (DENV-1 to DENV-4) transmitted primarily by *Aedes aegypti* and *Aedes albopictus* mosquitoes [1,2]. These mosquito-borne serotypes drive widespread epidemics, placing approximately 40% of the global population at risk of infection [1]. Dengue presents a clinical spectrum that spans from asymptomatic and mild febrile illness to severe forms, including fatal dengue hemorrhagic fever (DHF) and dengue shock syndrome (DSS) [3]. Dengue infection causes approximately 20,000 deaths annually, primarily among secondary infections that progress to DHF or DSS [4].

Globally, dengue represents a major and growing public health challenge. The global burden doubled over the past three decades, as the incidence and deaths increased from 26.45 million to 58.96 million cases and 14,315 to 29,075 deaths between 1990 and 2021, respectively [5]. According to the World Health Organization’s (WHO) global dengue surveillance data, 2024 recorded an unprecedented surge in dengue incidence, with more than 14.6 million reported cases and over 12,000 associated deaths across 100 countries [6]. Tropical and subtropical regions, specifically South Asia, Southeast Asia, Western Sub-Saharan Africa, and Tropical and Central Latin America, continue to experience the highest burden [5]. India accounts for a substantial proportion of the global dengue burden among the affected countries. The number of reported cases in India is increasing as a result of recurrent outbreaks and expanding geographic distribution [7]. Dengue hotspots have expanded across the central, western, and northern regions of India, now affecting 66.72% of the country’s territory [8].

Dengue fever was initially documented in India in 1956 in the Vellore district of Tamil Nadu [9]. The first recorded outbreak of DHF occurred in Calcutta (now Kolkata), West Bengal, in 1963 [10]. Several regions across the country, including newly affected areas, have reported dengue fever outbreaks ever since. The number of states and Union Territories (UTs) reporting dengue cases has increased from 8 (7 states and 1 UT) in 2000 to 35 (28 states and 7 UTs) at present [11]. Over the past two decades (2000-2009 and 2010-2019), dengue fever has substantially increased across India, marked by an elevenfold rise in reported cases [12]. According to the National Centre for Vector Borne Diseases Control (NCVBDC), around 1.1 million confirmed dengue cases and 1653 deaths were reported in India from 2019 to 2024 [13]. However, studies suggest these figures may be underestimates due to underreporting and misdiagnosis, with the actual annual burden potentially exceeding several million cases [14,15].

Dengue is a climate-sensitive disease, and temperature, rainfall, and relative humidity constitute the primary climatic factors that influence the life cycle, behavior, and distribution of the *Aedes* mosquito vectors and eventually the transmission of the disease [16,17]. Several spatial and climatic modelling studies have examined the influence of environmental drivers on dengue transmission using approaches such as spatial autocorrelation [18], spatial regression frameworks (e.g., simultaneous autoregressive models [SAR], conditional autoregressive [CAR], and Bayesian hierarchical models (BHM) [19], ecological niche modelling [20], and climate-disease time-series models [21]. Prior research from India and other endemic regions has highlighted the role of temperature, rainfall, humidity, and land-use characteristics in shaping dengue risk patterns across districts and over time. Rising temperatures, increased rainfall, and prolonged humidity create favourable conditions for the proliferation of *Aedes* mosquitoes and the transmission of dengue [22]. Warmer climates accelerate viral incubation within mosquitoes, while moderate rainfall sustains breeding sites, and extreme weather events, such as floods and hurricanes, generate new ones [23,24]. High humidity enhances mosquito survival and activity, whereas strong winds can limit dispersal and host contact [25]. Overall, these climatic changes are expanding dengue into new regions and complicating control efforts. In addition to climatic variability, the expansion of dengue in India has been actively driven by a complex interplay of factors, including unplanned urbanization, environmental changes, host-pathogen dynamics, and population-level immunological shifts [7]. These developments are driven by broader influences such as globalization of travel and trade, which facilitate the spread of pathogens and vectors, as well as climate change and evolving human behaviors [7].

Given India’s vast climatic diversity, encompassing tropical monsoon, hot arid, temperate, cold dry, and polar climates [26], understanding the spatial patterns of dengue incidence within different climatic contexts is critical for risk assessment and effective disease surveillance. However, despite substantial research on dengue epidemiology in India, nation-wide analyses that systematically examine the spatial distribution, clustering and risk assessment of dengue incidence across major climatic zones at the district level remain limited. Given the background, there is an urgent need to investigate the spatial heterogeneity of dengue incidence across India’s distinct climatic zones. This study provides a comprehensive analysis of the spatio-temporal dynamics of dengue incidence across diverse climate zones in India, offering insights into the epidemiological implications of climatic and demographic variability on dengue transmission.

## Methodology

### Study area and climate classification

**Fig 1:**
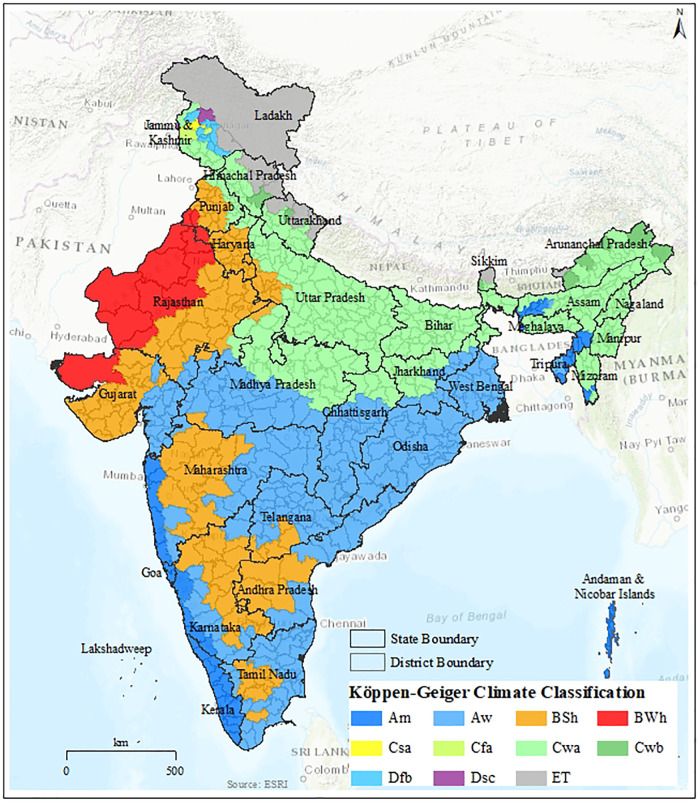
Geographical distribution of climatic zones in India, based on Köppen-Geiger Climate classification datasets (1980-2016). The map was developed using the licensed ArcGIS 10.4 software (Esri). The background base map shows topography. For more information about the base map services, visit http://goto.arcgisonline.com/maps/World_Topo_Map. Administrative boundary shapefiles were sourced from the Survey of India’s Administrative Boundary Database (https://onlinemaps.surveyofindia.gov.in/Digital_Product_Show.aspx).

The present study was conducted for the entire India, characterized by diverse climatic conditions and geographical variations. For the purpose of categorizing the dengue epidemiology based on the climate zones, the latest available high-resolution (1 kilometer) Köppen-Geiger Climate classification datasets (1980-2016) were used [27]. The Köppen-Geiger climate classification scheme has five primary categories (tropical, arid, temperate, cold, and polar climate) and 30 subtypes [27]. It divides India into five primary categories and 11 subtypes. The district boundaries, obtained from the Survey of India [28] were used to categorizing the climate zones; out of the 718 districts, 284 districts fall under temperate, dry winter, hot summer (Cwa) climate, 224 in tropical savannah (Aw), 122 in Arid, steppe, hot (BSh), 43 in tropical, rainforest (Am), 13 in the arid, desert, hot (BWh), 10 in polar, tundra (ET), 8 each in both temperate, dry winter, warm summer (Cwb) and cold, no dry season, warm summer (Dfb), 3 in temperate, no dry season, hot summer (Cfa), 2 in temperate, dry summer, hot summer (Csa) and one district in cold, dry summer, cold summer (Dsc) (Fig 1 and S1 Table).

### Epidemiological and population data

The epidemiological data cover reported dengue incidence in 718 districts across the States and UTs in India from 2010 to 2022. District-wise annual dengue incidence data were obtained from the NCVBDC, the central agency that collects and records data on the major vector-borne diseases (VBD), including dengue. In India, the dengue surveillance system is managed by the State government and the National Vector Borne Disease Control Program (NVBDCP); the health centers/hospitals report the confirmed laboratory cases of dengue to the district medical officer (DMO), which is forwarded to the state government, and onto NCVBDC, where it is systematically maintained and reviewed [7].

Population data were sourced from population projections derived from the 2011 Census of India [29]. The projections are generated using two distinct methodological approaches across states and union territories. For the seven north-eastern states (Arunachal Pradesh, Manipur, Meghalaya, Mizoram, Nagaland, Sikkim and Tripura), the projection uses a ‘mathematical method’ that examines past and present population data to capture trends, as these states collectively account for only about 1% of India’s total population. The projection applies the ‘cohort component method’ for the remaining 21 states and the UT of New Delhi, which evaluates annual birth cohorts by analyzing key demographic factors such as fertility, mortality and migration [29].

### Temporal trend analysis

Prais-Winsten regression was applied to assess temporal trends of dengue incidence rate (per 100,000) across climate zones, while accounting for serial autocorrelation [30–32]. Annual Increment Rates (AIR), along with their 95% confidence intervals (CI) and corresponding p-values, were used to classify trends as increasing, decreasing, or stationary. Trends with non-significant p-values were deemed stationary; significant p-values, combined with the direction of the AIR (+/-), indicated either an increasing (+AIR) or decreasing (-AIR) trend. AIR estimates were defined as [33]:


$$\begin{array}{c} AIR=\;\left[-\text{1}\;+\;(\text{1}{\text{0}}^{\beta })\right]*\text{100} \end{array}$$ (1)

where β is the natural-log coefficient obtained from the Prais-Winsten regression model. All analyses and visualizations were conducted using the ‘*prais*’ [34] and ‘*ggplot2*’ [35] packages in R (version 4.4.1, R Foundation for Statistical Computing, Vienna, Austria).

### Kruskal-Wallis test and Post-Hoc comparison

Initially, the major climate zones identified across the Indian districts were combined into three broader categories- Tropical (Z1), Temperate (Z2), and Arid (Z3). Cold and Polar climate regions were excluded from the analysis due to the negligible number of reported dengue cases in these areas during the study period. Normality of the data was assessed using the Shapiro-Wilk test, which indicated non-normality (P < 0.001). Therefore, the year-wise dengue cases were compared over three climate zones (Z1, Z2 and Z3) using a non-parametric test, the Kruskal-Wallis test, followed by a post hoc analysis (Pairwise Wilcoxon rank-sum test) to identify which climatic zones differed significantly from one another (Z1 vs. Z2, Z1 vs. Z3 and Z2 vs. Z3). Bonferroni adjustment was applied to the p-values for multiple comparisons to control for Type I error and ensure the robustness of the results [36,37].

### Spatial autocorrelation analysis

Global Moran’s I statistic was applied to evaluate the spatial autocorrelation and the global pattern (clustered, dispersed, random) of dengue incidence among the 718 districts in India. Global Moran’s I measure spatial autocorrelation, which accounts for both the spatial locations of features and their associated attribute values simultaneously. The spatial pattern is assessed using the global Moran’s I index (−1.0 to +1.0), indicating clustering (>0), dispersion (<0), or randomness (=0) [38]. The formulae to calculate the global Moran’s I index can be defined as [39]:


$$\begin{array}{c} I=\frac{n\Sigma_{i,j}W_{ij}\;(x_{i}-\overset{―}{x})(x_{j}-\overset{―}{x})}{\Sigma_{i,j}W_{ij}{\Sigma_{i}(x_{i}-\overset{―}{x})}^{\text{2}}} \end{array}$$ (2)

where n is the number of spatial units (districts) by i and j; $x_{i}$ represents the observed dengue incidence rate in the district i; $\overset{―}{x}$ is the mean of the dengue incidence rate across all districts; $W_{ij}$ is the spatial weight that captures the spatial relationship between units i and j.

The presence of spatial clusters of dengue incidence at the district level between 2010 and 2022 was identified using Anselin’s Local Moran I (LISA) test statistic. The LISA cluster maps were used to illustrate spatial clusters, wherein the value at a given location is compared to the average values of its neighboring areas [40]. For the spatial unit (districts) i, LISA is computed as [40];


$$\begin{array}{c} I_{i}=\frac{x_{i}-\overset{―}{x}}{S^{\text{2}}}\sum_{j}W_{ij}(x_{j}-\overset{―}{x}) \end{array}$$ (3)

where $S^{\text{2}}$ is the variance of x. A positive or negative value of $I_{i}$ is interpreted as indicating spatial clustering of similar or dissimilar incidence rates, respectively. Statistical significance was assessed using z-scores and p-values, which indicate whether observed spatial clustering or outliers deviate from random expectations. Based on these metrics, districts were classified as high-high (HH), low-low (LL), high-low (HL), low-high (LH), or non-significant [41]. The high value areas can be identified as hot spots (HH cluster) if the areas have a high-value neighborhood, and an LL cluster if the neighboring value is low [42]. A first-order queen contiguity spatial weights matrix was constructed, whereby districts sharing either a common boundary or a vertex were defined as neighbors. This specification is appropriate for administrative units with heterogeneous geometries and allows both edge and corner adjacencies to influence spatial structure [43]. All layers were converted into spatial layers for analysis and subsequently mapped using licensed ArcGIS software (version: 10.4; https://desktop.arcgis.com).

## Results

### Descriptive analysis

The epidemiological data across various climate zones, highlighting regional disparities in dengue incidence, incidence rate and population proportions, is shown in Table 1. Tropical savannah climatic zone (Aw) exhibits the highest disease burden, with a mean annual incidence rate of 2,219.68 per 100,000 population and a peak of 4,066.35 per 100,000 population, accounting for 44,906.77 mean annual cases across 32.57% of the total population. Despite covering a larger proportion of the population (36.91%), the temperate dry winter zone (Cwa) reported notably fewer mean annual cases (22,188.54) and a lower mean annual incidence rate of 1,831 per 100,000 population, suggesting that population size alone does not drive transmission. The hot arid zone (BSh), covering 21.13% of the population, reported 29,730 mean annual cases with a mean annual incidence rate of 1,261 per 100,000 population.

**Table 1:** Descriptive analysis of mean annual dengue incidence by climate zones across the Indian Districts from 2010 to 2022.

Climate zones	District Counts	Total Population Proportion (%) (Census 2011-2022 Projected)	Mean Annual Incidence Rate (per 100,000 people)	Peak Mean Annual Incidence Rate (per 100,000 people)	Mean Annual Cases	Peak Annual Mean Cases
Am	43	6.65	973.64	2,197.21	8,572.38	16,132.00
Aw	224	32.57	2,219.68	4,066.35	44,906.77	93,111.00
BSh	122	21.13	1,261.32	2,41786	29,729.77	54,100.33
BWh	13	1.78	355.18	806.48	3,514.54	8,562.67
Cfa	3	0.08	0.99	3.56	5.00	18.00
Csa	2	0.20	0.02	0.09	0.31	1.33
Cwa	284	36.91	1,831.15	4,136.79	22,188.54	52,369.33
Cwb	8	0.11	2.21	9.60	7.00	30.33
Dfb	8	0.36	18.43	62.00	64.08	219.67
Dsc	1	0.03	0.00	0.00	0.00	0.00
ET	10	0.18	1.50	5.20	4.23	14.33

**Am:** Tropical, monsoon; **Aw**: Tropical, savannah; **BSh**: Arid, steppe, hot; **BWh**: Arid, desert, hot; **Cfa**: Temperate, no dry season, hot summer; **Csa**: Temperate, dry summer, hot summer; **Cwa**: Temperate, dry winter, hot summer: **Cwb**: Temperate, dry winter, warm summer; **Dfb**: Cold, no dry season, warm summer; **Dsc**: Cold, dry summer, cold summer; **ET**: Polar, tundra; **Mean Annual Incidence Rate (per 100,000 people):** Annual incidence rates were calculated for each district as ([cases ÷ projected population] × 100,000) and averaged across all 13 study years (2010-2022) and districts within each climate zone; **Peak Mean Annual Incidence Rate (per 100,000 people):** The three highest annual incidence rates during 2010-2022 were identified for each climate zone and averaged; **Mean Annual Cases:** The total number of cases across all districts within each climate zone was summed for each year, and then averaged over the study years; **Peak Annual Mean Cases:** The total number of cases across all districts within each climate zone was summed for each year, and then averaged across the three peak years.

S1 Fig shows the monthly distribution of dengue incidence in India from 2010-2022, revealing a clear seasonal pattern. Dengue transmission is consistently low during the winter and early summer months (January to May) across most States, followed by a marked rise that begins in June and peaks between August and October.

In contrast, temperate zones such as Cfa, Csa, Cwb, and cold/polar zones such as Dfb, Dsc, and ET show markedly lower incidence rates and minimal contributions to overall caseloads. The Cfa and Csa zones reported negligible mean incidence rates of 0.99 and 0.02 per 100,000 population, respectively, corresponding to only 5 and 0.31 average annual cases. The Dsc zone shows zero recorded dengue incidence during the study period, while ET and Cwb reported extremely low incidence rates of 1.50 and 2.21, respectively (Table 1).

### Temporal trends of dengue cases across climate zones

**Fig 2:**
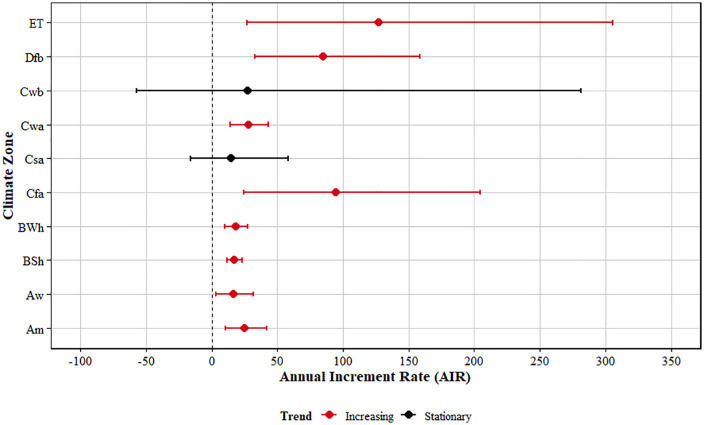
Trends in dengue incidence rates (per 100,000) across Köppen-Geiger climate zones in India from 2010-2022. Annual Increment Rates (AIR) and corresponding 95% confidence intervals are displayed for each climate zone. Significant positive trends are highlighted in red, and non-significant (stationary) trends in grey; the vertical dashed line represents the zero-change reference.

Fig 2 shows the temporal trends in total dengue cases reported between 2010 and 2022 across ten different Köppen climate classifications in India. Prais-Winsten regression was used to assess temporal trends and the annual increment rate in each zone.

The analysis shows consistently increasing dengue incidence across all Köppen-Geiger climate zones, with every zone demonstrating a statistically significant positive AIR, except for Csa and Cwb. The strongest increasing trends were observed in ET (AIR: 126.9%; 95% CI: 27.00-305.38; p: 0.01), Cfa (AIR: 94.8%; 95% CI: 24.59-204.67; p: 0.01), and Dfb (AIR: 85.1%; 95% CI: 32.65-158.35; p < 0.001). However, the wide confidence intervals in these zones reflect their small district counts, low case numbers, and minimal population proportions (Table 1), and findings should be interpreted with caution. Cwa (AIR: 28.03%; 95% CI: 14.43-43.25), Am (AIR: 25.34%; 95% CI: 10.82-41.76), BWh (AIR: 18.56%; 95% CI: 10.25-27.49), BSh (AIR: 17.46%; 95% CI: 11.73-23.48) and Aw (16.83%; 95% CI: 3.58-31.77), also demonstrated a statistically significant upward trend, with comparatively narrower confidence intervals indicating more stable trend estimates.

### Climatic zone differences in annual disease incidence

Table 2 presents a longitudinal analysis of dengue incidence rates across three climate zones, tropical (Z1), temperate (Z2), and arid (Z3), from 2010 to 2022, using Kruskal-Wallis tests and pairwise Wilcoxon comparisons to assess the statistical differences. Across all years, the Kruskal-Wallis test yielded highly significant results (p < 0.001), indicating significant differences in dengue incidence among the three categories. Median dengue incidence in the tropical zone consistently exceeded those in the temperate zone, with notable increases from 1 (0, 20.5) in 2010 to 97 (15, 322.5) in 2019, and remaining elevated through 2022.

**Table 2:** Year-wise comparison of dengue cases across different climate zones in India.

Year	Summary statistics	Climate Zone			Kruskal-Wallis Test (P value)	Post-hoc Comparison^#^ (pairwise Wilcoxon test)		
		Tropical (Z1) N = 267	Temperate (Z2) N = 296	Arid (Z3) N = 136		Z1/Z2	Z1/Z3	Z2/Z3
2010	Median (Q1, Q3)	1 (0, 20.5)	0 (0, 2)	14 (0.75, 43.25)	<0.001*	<0.001*	<0.001*	<0.001*
2011	Median (Q1, Q3)	3 (0, 15.5)	0 (0, 0)	6 (0, 25.75)	<0.001*	<0.001*	0.127	<0.001*
2012	Median (Q1, Q3)	8 (0, 96)	0 (0, 4)	19.5 (0, 70)	<0.001*	<0.001*	0.378	<0.001*
2013	Median (Q1, Q3)	21 (0, 110.5)	1 (0, 12)	40.5 (5, 165.75)	<0.001*	<0.001*	0.020*	<0.001*
2014	Median (Q1, Q3)	14 (0, 76.5)	0 (0, 3)	14 (2, 48.75)	<0.001*	<0.001*	1.000	<0.001*
2015	Median (Q1, Q3)	22 (2, 136.5)	1.5 (0, 25)	70.5 (10, 195.75)	<0.001*	<0.001*	<0.001*	<0.001*
2016	Median (Q1, Q3)	35 (3, 161)	8 (0, 50.75)	64.5 (14.75, 214.75)	<0.001*	<0.001*	0.031*	<0.001*
2017	Median (Q1, Q3)	37 (6, 330.5)	8 (1, 33.25)	100 (16, 383.75)	<0.001*	<0.001*	0.130	<0.001*
2018	Median (Q1, Q3)	49 (8, 195)	6 (1, 32.25)	72 (16., 225.5)	<0.001*	<0.001*	0.678	<0.001*
2019	Median (Q1, Q3)	97 (15, 322.5)	11 (2, 62)	186.5 (52, 434)	<0.001*	<0.001*	<0.001*	<0.001*
2020	Median (Q1, Q3)	17 (3, 58.5)	2 (0, 8)	40.5 (15, 122.25)	<0.001*	<0.001*	<0.001*	<0.001*
2021	Median (Q1, Q3)	94 (18, 310.5)	8 (1, 116.25)	233 (98.75, 502.75)	<0.001*	<0.001*	<0.001*	<0.001*
2022	Median (Q1, Q3)	87 (14, 278)	25.5 (3, 154.5)	155 (58, 327.5)	<0.001*	<0.001*	<0.001*	<0.001*

**N**: total number of districts**;** *Significant at p < 0.05; **^#^**P-values adjusted by Bonferroni correction

The arid zone frequently demonstrates the highest medians, particularly in later years, reaching 233 (98.75, 502.75) in 2021 and 155 (58, 327.5) in 2022, surpassing the Z1 zone in peak periods. The pairwise comparisons also exhibited significant statistical differences between Z1 and Z3 in most of the years, suggesting differences in disease burden during those periods. In contrast, the difference between Z1 and Z2 is statistically significant in every year (p < 0.001), highlighting a persistent disparity in disease incidence between tropical and temperate climates. These findings underscore the dominant influence of climatic conditions on disease patterns, with tropical (Z1) and arid (Z3) districts experiencing higher and more variable burdens than the districts under the temperate (Z2) zone.

### Spatial patterns of epidemic temporal indices

**Fig 3:**
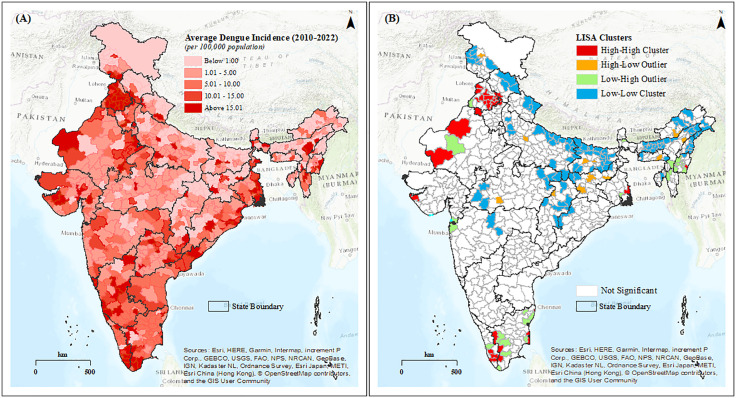
(A) Average dengue incidence (per 100,000) across the Indian districts from 2010-2022, and (B) spatial clusters of dengue incidence rate identified using Anselin’s Local Moran’s I. The map was developed using the licensed ArcGIS 10.4 software (Esri). The background base map shows topography. For more information about the base map services, visit http://goto.arcgisonline.com/maps/World_Topo_Map. Administrative boundary shapefiles were sourced from the Survey of India’s Administrative Boundary Database (https://onlinemaps.surveyofindia.gov.in/Digital_Product_Show.aspx).

A statistically significant positive spatial autocorrelation (Moran’s I = 0.06, Z = 2.95, P < 0.001) was observed across the districts, indicating a clustered spatial distribution of dengue incidence rate rather than a random pattern. Fig 3 (A) and (B) show the average dengue incidence (per 100,000) across the Indian districts from 2010 to 2022, and spatial clusters of dengue incidence rate identified using Anselin’s Local Moran’s I, respectively. The district-level local spatial clustering of dengue incidence rates across various climate zones in India is presented in S2 Table. Notable clusters were observed in several climate zones. Arid, temperate and tropical zones reported statistically significant clusters of high values, with the majority being in BSh (arid) and Cwa (temperate) zones (9 districts each). S3 Table further shows the list of 31 districts classified as significantly HH clusters. Notably, Punjab accounted for 12 such districts, highlighting a concentrated clustering in this region. Tamil Nadu (5 districts), Haryana (3), and Kerala (3) also showed localized clustering. Individual high-value clusters were also detected in Rajasthan, West Bengal, Chandigarh, Gujarat, Himachal Pradesh, and Manipur.

## Discussion

The key findings highlight considerable heterogeneity in dengue incidence across climate zones, with tropical savanna, temperate-dry winter-hot summer, and arid-steppe-hot zones bearing the highest burden, which together cover more than 85% of India’s geographic area. Prais-Winsten regression analysis indicates consistently increasing dengue incidence across all Köppen-Geiger climate zones, with every zone demonstrating a statistically significant positive AIR, except for Csa and Cwb. Kruskal-Wallis and pairwise comparison tests confirmed persistent and significant disparities between tropical, temperate, and arid regions. A statistically significant positive spatial autocorrelation indicated a clustered distribution of dengue incidence across Indian districts from 2010 to 2022, with Anselin’s Local Moran’s I identifying 31 high-high clusters predominantly in BSh and Cwa zones (9 districts each), including concentrated clustering in semi-arid Punjab-Haryana, and additional hotspots in humid Tamil Nadu-Kerala states.

Tropical and subtropical regions with warm, humid climates account for the majority of global dengue cases, with South and Southeast Asia emerging as major hotspots bearing the highest disease burden [44]. Here, the tropical and subtropical zones, Aw, Cwa, and BSh, recorded the highest mean annual incidence rates (2,219.68, 1,831.15 and 1,261.32 per 100,000, respectively), indicating a strong link between warm, wet climates and elevated dengue transmission. These high-burden climate zones are characterized by distinct seasonal patterns: Aw experiences a rainy summer and dry winter, Cwa features hot summers with dry winters, while BSh is marked by an arid environment with high temperatures, intense sunlight, minimal and irregular rainfall, and occasional torrential flooding [45]. A recent study from South and South-East Asian countries reported that warm-wet events were significantly associated with an increased dengue fever risk [44]. In India, warm-wet events occur frequently in the highly populated Cwa and BSh zones, which encompass a substantial share of the national population (32.57% and 36.91%, respectively), further amplifying their contribution to the overall disease burden. High temperatures and seasonal precipitation, coupled with dense human habitation and urban expansion, create optimal conditions for vector proliferation and disease transmission [7]. Furthermore, Cwa and BSh zones exhibited high and rising incidence rates, indicating dengue risk is not confined to tropical humid zones but is extending into adjacent temperate and dry climatic regimes. Similar emerging evidence across the globe reports that dengue is increasingly reported in semi-arid and temperate zones [46,47]. The differences in caseload in these climates underscore the complex interplay between environmental conditions and vector ecology. The findings of spatial heterogeneity support previous research that emphasizes warm temperatures and seasonal rainfall are increasing *Aedes* mosquito activity and dengue transmission [7,17].

Temporal trend analyses further identified the expanding geographical scope of dengue transmission in India. Notably, climate zones previously considered marginal for dengue, such as the polar (tundra) and cold (no dry season, warm summer) regions, now demonstrate the highest AIR across the study period, indicating a drastic shift in disease transmission patterns. This suggests a potential altitudinal and latitudinal expansion of vector habitats, likely driven by induced warming and altered precipitation patterns [48]. Climate change has resulted in warmer temperatures at higher altitudes, facilitating the establishment of *Ae. aegypti* and *Ae. albopictus* populations in regions exceeding 1500 meters above sea level, which were previously unsuitable for these vector species [48]. Rising minimum temperatures have contributed to increased survival and reproductive potential of *Aedes* mosquitoes [49]. In India, the spatiotemporal spread of dengue across geographical regions was not uniform—while dengue infections were reported across most Indian states by the 2000s, the hilly North-Eastern state of Mizoram did not report dengue cases until 2014 [50]. Our group has shown an annual increase of 0.02 °C per year in the minimum temperature over the past 30 years in Mizoram, the likely effect of deforestation and climate change, which could have facilitated the breeding of *Aedes* [50].

Concurrently in South Asia, over the past two decades, regions traditionally characterized by high rainfall have experienced a decline in precipitation, whereas areas with historically low rainfall have observed an increase [51]. These shifts in climatic patterns may further influence the distribution and dynamics of *Aedes* populations. Furthermore, the significantly increasing trends in BSh, BWh, and Cwa zones point toward growing dengue vulnerability in hot, dry, and temperate climates. BSh and BWh, traditionally considered less favorable for mosquito breeding [52] due to low humidity and water scarcity, are now experiencing more sporadic rainfall [53] and urban water storage, which creates microhabitats suitable for mosquito proliferation [54].

The Kruskal-Wallis test, followed by post-hoc pairwise Wilcoxon comparisons, revealed consistent differences in the dengue incidence across climate zones over the 13-year period (2010-2022). Across the study period, there is a statistically significant variation in the distribution of dengue cases among tropical, temperate, and arid zones. Notably, tropical and arid regions exhibited markedly higher median values compared to temperate regions, especially in the latter half of the study period (2015 onwards). Consistently, Mandal B. et al. (2024) reported that tropical regions such as Andhra Pradesh and Chhattisgarh exhibit the strongest positive trends in dengue incidence across the study period (2003-2022) [8]. The higher and increasingly elevated dengue incidence in these regions may be indicative of climatic suitability or population density contributing to greater risk or case burden [55–57]. Additionally, sustained positive trends in dengue cases over the last two decades in the western state of Goa [8] are likely attributable to rapid urbanization and infrastructure expansion [58] driven by growing tourism demands.

The presence of a statistically significant positive spatial autocorrelation suggests that dengue incidence across Indian districts exhibits a non-random, clustered spatial distribution. The spatial clusters identified using LISA analysis reveal that HH clusters are not uniformly distributed but are concentrated within specific climatic zones. Notably, the highest concentrations of significant dengue clusters were observed in the BSh and Cwa zones, each with nine districts identified as high-value clusters (S2 Table). Punjab reported the highest number of high-cluster districts for dengue incidence, and out of the 12 high-cluster districts, 8 are under the arid steppe (BSh) climate zone. Recent hydroclimatic trends indicate statistically significant increases in both annual and wet season rainfall across key locations in the hot arid region, particularly in Punjab, suggesting a notable shift in the regional precipitation patterns [59]. Increased rainfall in areas previously considered less susceptible can facilitate the geographic expansion of *Aedes* mosquitoes, enabling the spatial expansion of dengue into new regions [60]. Additionally, the extreme temperature variations, coupled with episodic rainfall events, can shorten the mosquito’s life cycle and accelerate viral replication, thereby increasing transmission efficiency during peak seasons [49].

Tamil Nadu showed significant clusters in the Aw climate zone, which is characterized by consistently high temperatures (all months ≥ 18 °C) and a distinct dry season, with the driest winter month receiving less than 60 mm of rainfall [61]. This climate is conducive to dengue transmission; minimum temperatures between 9.8 °C and 24.5 °C in tropical savanna (Aw) zones is associated with the increased dengue risk [62]. Climatic studies from Tamil Nadu show variables like rainfall and temperature significantly influence dengue incidence [63]. Urban heat island (UHI) effects, particularly in built-up areas, elevate surface temperatures [64,65], creating warmer microclimates that enhance mosquito survival and dengue transmission [66].

Additionally, districts classified under the Am (Kerala), BWh (Haryana and Rajasthan), and Cwa (Punjab, Haryana, Chandigarh, Himachal Pradesh and Manipur) climate zones have also reported significantly high dengue clusters. Tropical monsoon (Am) climate type is characterized by consistently high temperatures throughout the year (≥ 18°C) and high total annual precipitation, resulting in persistently humid conditions, and the Cwa climate zone is characterized by hot summers (average temperature of the warmest month ≥ 22 °C) and mild, dry winters (coldest month between −3 °C and 18 °C), with a distinct monsoonal rainfall pattern where most precipitation occurs in summer [61]. These distinct climatic conditions play a critical role in shaping the environmental suitability for VBDs like dengue, particularly through their influence on mosquito breeding and virus transmission dynamics.

This study has some limitations. First, the dengue data used are only available at a district level, which prevents analysis at more detailed geographic units (micro levels). As a result, local variations in dengue patterns may not be fully captured. Second, the use of annual data constrained our ability to assess lagged or short-term dynamics, which typically require district-level monthly or weekly time series. Third, case locations reflect where cases were reported or treated, not necessarily where infections were acquired, introducing a degree of spatial misclassification inherent to surveillance-based datasets. Fourth, the use of Köppen-Geiger climate data (1980-2016) with dengue data extended up to 2022 may introduce slight temporal variation, as some climatic zones may have experienced gradual shifts over time. Additionally, in India, dengue cases are likely underreported [14,15], so the true extent of the disease remains unclear. Finally, assigning districts to specific climatic zones poses challenges, as many districts span transitional areas with overlapping climatic characteristics.

## Conclusions

Overall, dengue epidemiology in India appears to be undergoing a phase of climatic and geographic transformation. The observed shifts toward semi-arid and arid regions, along with intensifying incidence in traditionally low-risk zones, reflect broader trends associated with climate variability. They also highlight the inadequacy of current risk models that are primarily based on historical tropical transmission zones. Public health policies and vector control strategies must adapt to this changing landscape by incorporating climate-informed surveillance, region-specific interventions, and predictive modelling tools to prevent future outbreaks.

## Supporting information

S1 Table Description of the different Köppen-Geiger Climate classes and sub-types in India. (DOCX)

S1 Fig State-wise monthly climatology of dengue incidence in India, 2010-2022. To improve visual interpretability, dengue incidence was visualized using a natural log transformation [ln(x + 1)] to account for zero values and right-skewness.
(DOCX)

S2 Table District-level local spatial autocorrelation analysis results of dengue incidence rate in different climate zones of India, 2010-2022. (DOCX)

S3 Table Districts identified as statistically significant high-value clusters (High-High) using Local Moran’s I (LISA) spatial autocorrelation analysis. (DOCX)

## References

[1] Pourzangiabadi M, Najafi H, Fallah A, Goudarzi A, Pouladi I. Dengue virus: Etiology, epidemiology, pathobiology, and developments in diagnosis and control - A comprehensive review. Infect Genet Evol. 2025;127:105710. doi: 10.1016/j.meegid.2024.105710 39732271

[2] Kakarla SG, Bhimala KR, Kadiri MR, Kumaraswamy S, Mutheneni SR. Dengue situation in India: Suitability and transmission potential model for present and projected climate change scenarios. Sci Total Environ. 2020;739:140336. doi: 10.1016/j.scitotenv.2020.140336 32758966

[3] Bhatt P, Sabeena SP, Varma M, Arunkumar G. Current Understanding of the Pathogenesis of Dengue Virus Infection. Curr Microbiol. 2021;78(1):17–32. doi: 10.1007/s00284-020-02284-w 33231723 PMC7815537

[4] Pang X, Zhang R, Cheng G. Progress towards understanding the pathogenesis of dengue hemorrhagic fever. Virol Sin. 2017;32(1):16–22. doi: 10.1007/s12250-016-3855-9 27853992 PMC6702245

[5] Zhang W-X, Zhao T-Y, Wang C-C, He Y, Lu H-Z, Zhang H-T, et al. Assessing the global dengue burden: Incidence, mortality, and disability trends over three decades. PLoS Negl Trop Dis. 2025;19(3):e0012932. doi: 10.1371/journal.pntd.0012932 40072961 PMC11925280

[6] WHO. Dengue. https://www.who.int/news-room/fact-sheets/detail/dengue-and-severe-dengue

[7] Mutheneni SR, Morse AP, Caminade C, Upadhyayula SM. Dengue burden in India: recent trends and importance of climatic parameters. Emerg Microbes Infect. 2017;6(8):e70. doi: 10.1038/emi.2017.57 28790459 PMC5583666

[8] Mandal B, Mondal S. Unveiling spatio-temporal mysteries: A quest to decode India’s dengue and malaria trend (2003-2022). Spatial and Spatio-temporal Epidemiology. 2024;51:100690. doi: 10.1016/J.SSTE.2024.100690 39615969

[9] Carey DE, Myers RM, Reuben R, Rodrigues FM. Studies on dengue in Vellore, South India. Am J Trop Med Hyg. 1966;15(4):580–7. doi: 10.4269/ajtmh.1966.15.580 5949559

[10] Aikat BK, Konar NR, Banerjee G. Haemorrhagic Fever In Calcutta Area. Indian J Med Res. 1964;52:660–75. 14195506

[11] Prajapati AK, Singh NP, Jain PK, Srivastava DK, Prajapati R. Dengue in India: An Overview. National Journal of Community Medicine. 2022;13: 49–57. doi:10.5455/njcm.20211204035455

[12] Baruah K, Arora N, Sharma H, Katewa A. Dengue in India: Temporal and Spatial Expansion in Last Two Decades. Journal of Medical Arthropodology and Public Health. 2021;1:15–32.

[13] NCVBDC. Dengue Situation in India. National Center for Vector Borne Diseases Control, Ministry of Health & Family Welfare, Government of India. 2025. https://ncvbdc.mohfw.gov.in/index4.php?lang=1&level=0&linkid=431&lid=3715

[14] Das S, Sarfraz A, Jaiswal N, Das P. Impediments of reporting dengue cases in India. J Infect Public Health. 2017;10(5):494–8. doi: 10.1016/j.jiph.2017.02.004 28262571

[15] Shepard DS, Halasa YA, Tyagi BK, Adhish SV, Nandan D, Karthiga KS, et al. Economic and disease burden of dengue illness in India. The American Journal of Tropical Medicine and Hygiene. 2014;91:1235. doi: 10.4269/AJTMH.14-0002 25294616 PMC4257651

[16] Hussain SSA, Dhiman RC. Distribution Expansion of Dengue Vectors and Climate Change in India. Geohealth. 2022;6(6):e2021GH000477. doi: 10.1029/2021GH000477 35769847 PMC9210256

[17] Bhatia S, Bansal D, Patil S, Pandya S, Ilyas QM, Imran S. A retrospective study of climate change affecting dengue: evidences, challenges and future directions. Frontiers in Public Health. 2022;10:884645. doi: 10.3389/FPUBH.2022.884645 35712272 PMC9197220

[18] Mahato RK, Htike KM, Sornlorm K, Koro AB, Yadav RK, Kafle A, et al. Spatial autocorrelation of environmental factors influencing dengue outbreaks using Moran’s I: A study from Nepal (2020-2023). PLoS One. 2025;20(6):e0324798. doi: 10.1371/journal.pone.0324798 40465649 PMC12136339

[19] Aswi A, Cramb SM, Moraga P, Mengersen K. Bayesian spatial and spatio-temporal approaches to modelling dengue fever: a systematic review. Epidemiol Infect. 2018;147:e33. doi: 10.1017/S0950268818002807 30369335 PMC6518570

[20] Ashby J, Moreno-Madriñán M, Yiannoutsos C, Stanforth A. Niche Modeling of Dengue Fever Using Remotely Sensed Environmental Factors and Boosted Regression Trees. Remote Sensing. 2017;9(4):328. doi: 10.3390/rs9040328

[21] Alam KE, Ahmed MJ, Chalise R, Rahman MA, Mathin TT, Bhuiyan MIH, et al. Time series analysis of dengue incidence and its association with meteorological risk factors in Bangladesh. PLoS One. 2025;20(8):e0323238. doi: 10.1371/journal.pone.0323238 40824998 PMC12360610

[22] Abbasi E. Global expansion of Aedes mosquitoes and their role in the transboundary spread of emerging arboviral diseases: A comprehensive review. IJID One Health. 2025;6:100058. doi: 10.1016/j.ijidoh.2025.100058

[23] Kamiya T, Greischar MA, Wadhawan K, Gilbert B, Paaijmans K, Mideo N. Temperature-dependent variation in the extrinsic incubation period elevates the risk of vector-borne disease emergence. Epidemics. 2020;30:100382. doi: 10.1016/j.epidem.2019.100382 32004794

[24] Alkhaldy I. Modelling the association of dengue fever cases with temperature and relative humidity in Jeddah, Saudi Arabia-A generalised linear model with break-point analysis. Acta Trop. 2017;168:9–15. doi: 10.1016/j.actatropica.2016.12.034 28069326

[25] Gui H, Gwee S, Koh J, Pang J. Weather Factors Associated with Reduced Risk of Dengue Transmission in an Urbanized Tropical City. Int J Environ Res Public Health. 2021;19(1):339. doi: 10.3390/ijerph19010339 35010600 PMC8751148

[26] Krishnan R, Gnanaseelan C, Sanjay J, Swapna P, Dhara C, Sabin TP. Introduction to climate change over the Indian region. Assessment of climate change over the Indian region: A report of the Ministry of Earth Sciences (MoES), Government of India. 2020. 1–20. doi: 10.1007/978-981-15-4327-2_1/TABLES/5

[27] Beck HE, Zimmermann NE, McVicar TR, Vergopolan N, Berg A, Wood EF. Present and future Köppen-Geiger climate classification maps at 1-km resolution. Sci Data. 2018;5:180214. doi: 10.1038/sdata.2018.214 30375988 PMC6207062

[28] Ministry of Science and Technology. Survey of India. https://onlinemaps.surveyofindia.gov.in/Digital_Product_Show.aspx 2025.

[29] National Commission on Population. Population Projections for India and States 2011 - 2036. New Delhi: National Commission on Population Ministry of Health & Family Welfare. 2020. https://nhm.gov.in/New_Updates_2018/Report_Population_Projection_2019.pdf

[30] Böhm AW, Costa CDS, Neves RG, Flores TR, Nunes BP. Dengue incidence trend in Brazil, 2002-2012. Epidemiologia e Serviços de Saúde. 2016;25:725–33. doi: 10.5123/S1679-49742016000400006 27869966

[31] Mudelsee M. Trend analysis of climate time series: A review of methods. Earth-Science Reviews. 2019;190:310–22. doi: 10.1016/j.earscirev.2018.12.005

[32] S PJP, Christopher BW. Trend Estimators and Serial Correlation. Cowles Foundation for Research in Economics. 1954. https://cowles.yale.edu/sites/default/files/2023-05/s-0383.pdf

[33] Carvalho de Aquino É, Neves CM, Neto OLM. Trends in mortality due to road traffic accidents in the municipality of Goiânia, Brazil, 2006-2014*. Epidemiologia e Serviços de Saúde. 2018;26. 10.5123/s1679-49742018000400015 30570032

[34] Mohr FX. prais: Prais-Winsten Estimator for AR(1) Serial Correlation. CRAN: Contributed Packages. The R Foundation. 2015. doi: 10.32614/cran.package.prais

[35] Wickham H. ggplot2: Elegant Graphics for Data Analysis. 2nd ed. Cham: Springer International Publishing. 2016. doi: 10.1007/978-3-319-24277-4

[36] Lee S, Lee DK. What is the proper way to apply the multiple comparison test?. Korean J Anesthesiol. 2018;71(5):353–60. doi: 10.4097/kja.d.18.00242 30157585 PMC6193594

[37] Sedgwick P. Multiple significance tests: the Bonferroni correction. BMJ. 2012;344(jan25 4):e509–e509. doi: 10.1136/bmj.e509

[38] Yue Y, Sun J, Liu X, Ren D, Liu Q, Xiao X, et al. Spatial analysis of dengue fever and exploration of its environmental and socio-economic risk factors using ordinary least squares: A case study in five districts of Guangzhou City, China, 2014. Int J Infect Dis. 2018;75:39–48. doi: 10.1016/j.ijid.2018.07.023 30121308

[39] Moran PA. Notes on continuous stochastic phenomena. Biometrika. 1950;37(1–2):17–23. doi: 10.1093/BIOMET/37.1-2.17 15420245

[40] Anselin L. Local Indicators of Spatial Association—LISA. Geographical Analysis. 1995;27(2):93–115. doi: 10.1111/j.1538-4632.1995.tb00338.x

[41] Si X, Wang L, Mengersen K, Hu W. Epidemiological features of seasonal influenza transmission among 11 climate zones in Chinese Mainland. Infect Dis Poverty. 2024;13(1):4. doi: 10.1186/s40249-024-01173-9 38200542 PMC10777546

[42] Li R, Zhao X, Tian Y, Shi Y, Gu X, Wang S. Different responses of Japanese encephalitis to weather variables among eight climate subtypes in Gansu, China, 2005–2019. BMC Infectious Diseases. 2023;23:114. doi: 10.1186/S12879-023-08074-6 36823521 PMC9951518

[43] Gwitira I, Mukonoweshuro M, Mapako G, Shekede MD, Chirenda J, Mberikunashe J. Spatial and spatio-temporal analysis of malaria cases in Zimbabwe. Infect Dis Poverty. 2020;9(1):146. doi: 10.1186/s40249-020-00764-6 33092651 PMC7584089

[44] Wang Y, Chong KC, Ren C. Impact of compound warm and wet events on dengue fever infection in South and Southeast Asian countries. Environ Res. 2024;263(Pt 2):120091. doi: 10.1016/j.envres.2024.120091 39368600

[45] Rodrigues NCP, Lino VTS, Daumas RP, Andrade MK de N, O’Dwyer G, Monteiro DLM, et al. Temporal and Spatial Evolution of Dengue Incidence in Brazil, 2001-2012. PLoS One. 2016;11(11):e0165945. doi: 10.1371/journal.pone.0165945 27832129 PMC5104436

[46] Díaz-Castro M-L, Ortega-Rubio S-P. Relation between dengue and climate trends in the Northwest of Mexico. Tropical Biomedicine. 2017;34: 157–165. 33592994

[47] Nakhapakorn K, Tripathi NK. An information value based analysis of physical and climatic factors affecting dengue fever and dengue haemorrhagic fever incidence. Int J Health Geogr. 2005;4:13. doi: 10.1186/1476-072X-4-13 15943863 PMC1177981

[48] Abbasi E. The impact of climate change on travel-related vector-borne diseases: A case study on dengue virus transmission. Travel Med Infect Dis. 2025;65:102841. doi: 10.1016/j.tmaid.2025.102841 40118163

[49] Liu Z, Zhang Q, Li L, He J, Guo J, Wang Z, et al. The effect of temperature on dengue virus transmission by Aedes mosquitoes. Front Cell Infect Microbiol. 2023;13:1242173. doi: 10.3389/fcimb.2023.1242173 37808907 PMC10552155

[50] Karuppusamy B, Sarma DK, Lalmalsawma P, Pautu L, Karmodiya K, Balabaskaran Nina P. Effect of climate change and deforestation on vector borne diseases in the North-Eastern Indian State of Mizoram bordering Myanmar. The Journal of Climate Change and Health. 2021;2:100015. doi: 10.1016/j.joclim.2021.100015

[51] Jana S, Gogoi MM, Babu SS. Change in precipitation pattern over South Asia in response to the trends in regional warming and free-tropospheric aerosol loading. Sci Rep. 2024;14(1):14528. doi: 10.1038/s41598-024-64842-7 38914618 PMC11196666

[52] Iwamura T, Guzman-Holst A, Murray KA. Accelerating invasion potential of disease vector Aedes aegypti under climate change. Nat Commun. 2020;11(1):2130. doi: 10.1038/s41467-020-16010-4 32358588 PMC7195482

[53] Tayfur G. Discrepancy precipitation index for monitoring meteorological drought. Journal of Hydrology. 2021;597:126174. doi: 10.1016/j.jhydrol.2021.126174

[54] Newman EA, Feng X, Onland JD, Walker KR, Young S, Smith K, et al. Defining the roles of local precipitation and anthropogenic water sources in driving the abundance of Aedes aegypti, an emerging disease vector in urban, arid landscapes. Sci Rep. 2024;14(1):2058. doi: 10.1038/s41598-023-50346-3 38267474 PMC10808563

[55] Nakase T, Giovanetti M, Obolski U, Lourenço J. Population at risk of dengue virus transmission has increased due to coupled climate factors and population growth. Commun Earth Environ. 2024;5(1). doi: 10.1038/s43247-024-01639-6

[56] Wang Y, Zhao S, Wei Y, Li K, Jiang X, Li C, et al. Impact of climate change on dengue fever epidemics in South and Southeast Asian settings: A modelling study. Infect Dis Model. 2023;8(3):645–55. doi: 10.1016/j.idm.2023.05.008 PMC10333599 37440763

[57] Abbasi E. The impact of climate change on Aedes aegypti distribution and dengue fever prevalence in semi-arid regions: A case study of Tehran Province, Iran. Environ Res. 2025;275:121441. doi: 10.1016/j.envres.2025.121441 40118318

[58] Balasubramani K, Hussain SSA, Sawant SA, Govekar A, Telugu Prakash P, Naik A, et al. Dengue transmission dynamics in an urban setting in western India. PLoS Negl Trop Dis. 2026;20(3):e0013636. doi: 10.1371/journal.pntd.0013636 41871106 PMC13052988

[59] Machiwal D, Dayal D, Kumar S. Long-term rainfall trends and change points in hot and cold arid regions of India. Hydrological Sciences Journal. 2017;62(7):1050–66. doi: 10.1080/02626667.2017.1303705

[60] Dhimal M, Kramer IM, Phuyal P, Budhathoki SS, Hartke J, Ahrens B, et al. Climate change and its association with the expansion of vectors and vector-borne diseases in the Hindu Kush Himalayan region: A systematic synthesis of the literature. Advances in Climate Change Research. 2021;12(3):421–9. doi: 10.1016/j.accre.2021.05.003

[61] Kottek M, Grieser J, Beck C, Rudolf B, Rubel F. World Map of the Köppen-Geiger climate classification updated. metz. 2006;15(3):259–63. doi: 10.1127/0941-2948/2006/0130

[62] Damtew YT, Tong M, Varghese BM, Anikeeva O, Hansen A, Dear K, et al. Effects of high temperatures and heatwaves on dengue fever: a systematic review and meta-analysis. EBIOM. 2023. doi: 10.1016/j.ebiom.2023.104582 PMC10149186 37088034

[63] Chandy S, Ramanathan K, Manoharan A, Mathai D, Baruah K. Assessing effect of climate on the incidence of dengue in Tamil Nadu. Indian J Med Microbiol. 2013;31(3):283–6. doi: 10.4103/0255-0857.115640 23883717

[64] Bala RS, Sathyanarayanan S, Jeyaparakash ST. Understanding the relationship between Urban Heat Island and Urban Vegetation by reviewing three decades of satellite images for the Chennai Metropolitan Area. IOP Conf Ser: Earth Environ Sci. 2023;1210(1):012029. doi: 10.1088/1755-1315/1210/1/012029

[65] Bagyaraj M, Senapathi V, Karthikeyan S, Chung SY, Khatibi R, Nadiri AA, et al. A study of urban heat island effects using remote sensing and GIS techniques in Kancheepuram, Tamil Nadu, India. Urban Climate. 2023;51:101597. doi: 10.1016/j.uclim.2023.101597

[66] Wong GKL, Jim CY. Urban-microclimate effect on vector mosquito abundance of tropical green roofs. Building and Environment. 2017;112:63–76. doi: 10.1016/j.buildenv.2016.11.028

