# Supplementary material for: Spatial distribution and risk assessment of dengue incidence at district level across major climatic zones in India
Source: PLoS One. 2026 Jun 9;21(6):e0350325. doi: 10.1371/journal.pone.0350325 (PMC13249156; doi:10.1371/journal.pone.0350325)
Supplement: S1 Table — (DOCX) [file pone.0350325.s001.docx]

**S1 Table.** Description of the different Köppen-Geiger Climate classes and sub-types in India

| Sl. no. | Climate class | Climate sub-type | Description | No. of districts |
| --- | --- | --- | --- | --- |
| 1 | Tropical | Am | Tropical, monsoon | 43 |
|  |  | Aw | Tropical, savannah | 224 |
| 2 | Arid | BSh | Arid, steppe, hot | 122 |
|  |  | BWh | Arid, desert, hot | 13 |
| 3 | Temperate | Cfa | Temperate, no dry season, hot summer | 3 |
|  |  | Csa | Temperate, dry summer, hot summer | 2 |
|  |  | Cwa | Temperate, dry winter, hot summer | 284 |
|  |  | Cwb | Temperate, dry winter, warm summer | 8 |
| 4 | Cold | Dfb | Cold, no dry season, warm summer | 8 |
|  |  | Dsc | Cold, dry summer, cold summer | 1 |
| 5 | Polar | ET | Polar, tundra | 10 |
