# Supplementary material for: Spatial distribution and risk assessment of dengue incidence at district level across major climatic zones in India
Source: PLoS One. 2026 Jun 9;21(6):e0350325. doi: 10.1371/journal.pone.0350325 (PMC13249156; doi:10.1371/journal.pone.0350325)
Supplement: S1 Fig — To improve visual interpretability, dengue incidence was visualized using a natural log transformation [ln(x + 1)] to account for zero values and right-skewness. (DOCX) [file pone.0350325.s002.docx]

**
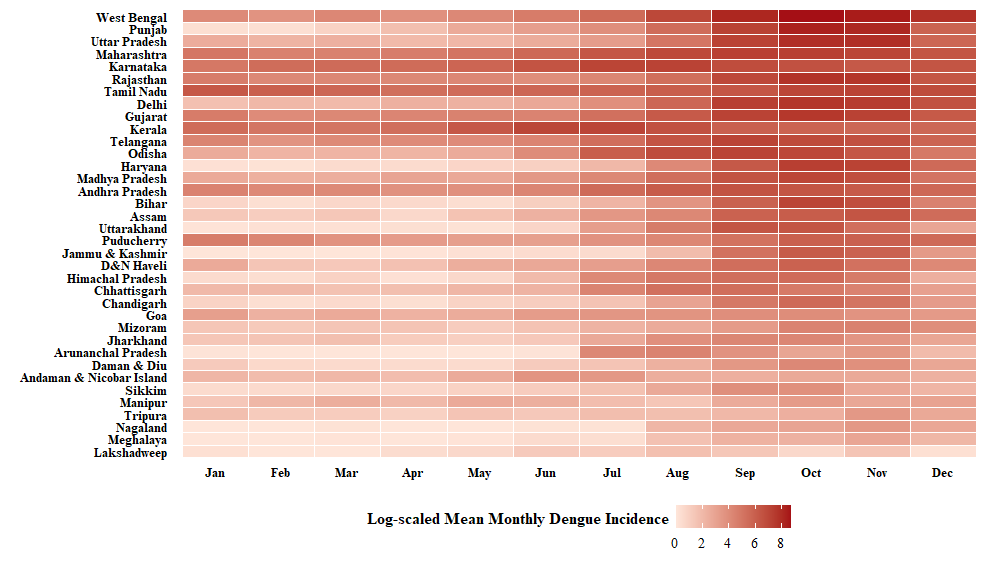
**

**S1 Fig.** State-wise monthly climatology of dengue incidence in India, 2010-2022. To improve visual interpretability, dengue incidence was visualized using a natural log transformation [ln(x + 1)] to account for zero values and right-skewness.
