# Supplementary material for: Spatial distribution and risk assessment of dengue incidence at district level across major climatic zones in India
Source: PLoS One. 2026 Jun 9;21(6):e0350325. doi: 10.1371/journal.pone.0350325 (PMC13249156; doi:10.1371/journal.pone.0350325)
Supplement: S2 Table — (DOCX) [file pone.0350325.s003.docx]

**S2 Table.** District-level local spatial clustering of dengue incidence rate in different climate zones of India, 2010-2022.

| Climate Zone | Number of districts | Dengue Incidence Rate | | | | |
| --- | --- | --- | --- | --- | --- | --- |
|  |  | **HH** | **HL** | **LH** | **LL** | **No significance** |
| Am | 42 | 3 | 1 | 3 | 4 | 31 |
| Aw | 223 | 7 | 2 | 7 | 12 | 195 |
| BSh | 122 | 9 | 0 | 3 | 2 | 108 |
| BWh | 13 | 3 | 0 | 2 | 0 | 8 |
| Cfa | 3 | 0 | 0 | 0 | 2 | 1 |
| Csa | 2 | 0 | 0 | 0 | 1 | 1 |
| Cwa | 284 | 9 | 8 | 10 | 77 | 180 |
| Cwb | 8 | 0 | 0 | 0 | 0 | 8 |
| Dfb | 8 | 0 | 1 | 0 | 4 | 3 |
| Dsc | 1 | 0 | 0 | 0 | 0 | 1 |
| ET | 10 | 0 | 0 | 0 | 6 | 4 |
| Total | 716 | 31 | 12 | 25 | 108 | 540 |

**HH**: High-High cluster; **HL**: High-Low cluster; **LH**: Low-High cluster; **LL**: Low-Low cluster; **Am**- Tropical, monsoon; **Aw**- Tropical, savannah; **BSh**- Arid, steppe, hot; **BWh**- Arid, desert, hot; **Cfa**- Temperate, no dry season, hot summer; **Csa**- Temperate, dry summer, hot summer; **Cwa**- Temperate, dry winter, hot summer: **Cwb**- Temperate, dry winter, warm summer; **Dfb**- Cold, no dry season, warm summer; **Dsc**: Cold, dry summer, cold summer **ET**: Polar, tundra.
