# Supplementary material for: Spatial distribution and risk assessment of dengue incidence at district level across major climatic zones in India
Source: PLoS One. 2026 Jun 9;21(6):e0350325. doi: 10.1371/journal.pone.0350325 (PMC13249156; doi:10.1371/journal.pone.0350325)
Supplement: S3 Table — (DOCX) [file pone.0350325.s004.docx]

**S3 Table.** Districts identified as statistically significant high-value clusters (High-High) of dengue using Local Moran’s I (LISA) spatial autocorrelation analysis.

| Sl.no. | Climate zone | District | State | Total no. in each state |
| --- | --- | --- | --- | --- |
| 1 | BSh | Amritsar | Punjab | 12 |
| 2 | BSh | Barnala | Punjab |  |
| 3 | BSh | Faridkot | Punjab |  |
| 4 | Cwa | Fatehgarh Sahib | Punjab |  |
| 5 | BSh | Jalandhar | Punjab |  |
| 6 | BSh | Ludhiana | Punjab |  |
| 7 | BSh | Moga | Punjab |  |
| 8 | Cwa | Patiala | Punjab |  |
| 9 | Cwa | Rupnagar | Punjab |  |
| 10 | BSh | Sangrur | Punjab |  |
| 11 | Cwa | Sahibzada Ajit Singh Nagar | Punjab |  |
| 12 | BSh | Tarn Taran | Punjab |  |
| 13 | Aw | Chennai | Tamil Nadu | 5 |
| 14 | Aw | Coimbatore | Tamil Nadu |  |
| 15 | Aw | Nagapattinam | Tamil Nadu |  |
| 16 | Aw | Theni | Tamil Nadu |  |
| 17 | Aw | Thenkasi | Tamil Nadu |  |
| 18 | Cwa | Ambala | Haryana | 3 |
| 19 | Cwa | Panchkula | Haryana |  |
| 20 | BWh | Sirsa | Haryana |  |
| 21 | Am | Kollam | Kerala | 3 |
| 22 | Am | Kottayam | Kerala |  |
| 23 | Am | Pathanamthitta | Kerala |  |
| 24 | BWh | Barmer | Rajasthan | 2 |
| 25 | BWh | Bikaner | Rajasthan |  |
| 26 | Aw | Kolkata | West Bengal | 2 |
| 27 | Aw | North 24 Parganas | West Bengal |  |
| 28 | Cwa | Chandigarh | Chandigarh | 1 |
| 29 | BSh | Devbhumi Dwarka | Gujarat | 1 |
| 30 | Cwa | Solan | Himachal Pradesh | 1 |
| 31 | Cwa | Tengnoupal | Manipur | 1 |

**BSh**: Arid, steppe, hot; **Cwa**- Temperate, dry winter, hot summer; **Aw**- Tropical, savannah; **BWh**- Arid, desert, hot; **Am**- Tropical, monsoon.
