# Supplementary material for: Spatial distribution and risk assessment of dengue incidence at district level across major climatic zones in India
Source: PLoS One. 2026 Jun 9;21(6):e0350325. doi: 10.1371/journal.pone.0350325 (PMC13249156; doi:10.1371/journal.pone.0350325)
Supplement: Attachment — Submitted filename: Authors Response Sheet.docx [file pone.0350325.s005.docx]

**AUTHOR’S RESPONSE**

**Manuscript:** Spatial Distribution and Risk Assessment of Dengue Incidence at District Level Across Major Climatic Zones in India

**Manuscript ID:** PONE-D-25-42541

We thank the Editor and the reviewers for their thorough evaluation of our manuscript and for their constructive and insightful comments. We have carefully considered all suggestions and have revised the manuscript extensively to address methodological, conceptual, and presentation-related concerns. Key revisions include the refinement of temporal trend analysis using Prais-Winsten regression to account for serial autocorrelation, clearer presentation of the study’s novelty and contribution within the existing literature, improved justification and explanation of the climatic framework, and a strengthened discussion section. We believe that these revisions have substantially improved the scientific rigour, clarity, and interpretability of the manuscript. We provide detailed point-by-point responses below.

**Reviewer #1:**

*This study is important and epidemiologically useful as it provides a comprehensive analysis of the spatiotemporal dynamics of dengue incidence in various climatic zones from India. The analysis that shows how climate and demographic variability has affected understanding the spatial patterns of its incidence and transmission is also important. These two aspects are of clinical and preventive value apply to other contexts.*

*The authors need to make all the data underlying the findings in their manuscript fully available upon request by the journal.*

We thank the reviewer for their careful review of our manuscript. All data underlying the findings reported in this study are included directly within the manuscript or provided in the supplementary information. Additional clarification or supporting materials will be supplied upon request by the journal.

**Reviewer #2:**

I have gone the manuscript thoroughly, and my review feedback as followed.

*1) The study based on retrospective dengue data spanning from 2010 to 2022. However, the authors' used linear regression for temporal trend analysis is a significant methodological flaw, as it fails to account for the serial correlation inherent in time-dependent data. A time series analysis would have been the correct approach.*

Thank you for highlighting the methodological concern regarding the use of linear regression for temporal trend analysis. We agree that distributed time-series analysis approaches are valuable for estimating short-term lagged effects and non-linear exposure-response relationships using high-resolution time series (e.g., daily or weekly data). However, our analysis uses annual, district-level dengue incidence (2010-2022). At this temporal resolution (13 annual observations per district), lagged and short-term effects cannot be meaningfully resolved using time-series analysis.

In response, we have revised our approach and now employ Prais-Winsten regression (PMID: 27869966, <https://cowles.yale.edu/sites/default/files/2023-05/s-0383.pdf>), which appropriately accounts for serial autocorrelation in time-dependent dengue incidence data (2010-2022). The analysis now estimates ‘Annual Increment Rates (AIR)’ with 95% confidence intervals and p-values to classify trends as increasing, decreasing, or stationary, following Antunes’ method.

All changes have been updated in the manuscript (Methodology: Temporal Trend Analysis).

*2) The study has a potential for bias because it did not account for the fact that the locations where infections were acquired and where patients received treatment were often different. This discrepancy could lead to an inaccurate representation of dengue incidence in specific areas.*

We agree with the reviewer that discrepancies between infection acquisition sites and treatment locations may introduce spatial misclassification. This is an inherent limitation of surveillance-based dengue datasets. At the same time, we note that this bias is likely limited because the administrative units used (districts) are geographically large, making it more probable that infection acquisition and treatment occur within the same district. However, we have now explicitly acknowledged this potential bias in the Limitations section and clarified that reported case locations may not fully represent the true spatial distribution of transmission.

*3) Conclusion in abstract is very short and understand to relate their results.*

Thank you for this observation. We have revised the concluding statement of the abstract to more clearly reflect and integrate our key temporal and spatial findings.

*4) A more appropriate approach would have been to analyze weather variability instead of climate change variability, given that the study used only 12 years of data. Climate change trends are typically assessed over much longer periods, such as 30 years or more.*

Thank you for this important observation. We would like to clarify that the primary focus of this study is climatic zonation, rather than assessing climate change trends. The Köppen-Geiger climate classification used in this study is derived from long-term climatological normals (1980-2016) (PMID: 30375988) and is intended to represent relatively stable, multi-decadal climatic regimes rather than short-term weather variability or recent climate change signals.

Our analysis examines how dengue incidence patterns vary across these established climate zones, using surveillance data from 2010 to 2022, without attempting to infer trends related to climate change. We have revised the manuscript to more explicitly distinguish between climate zone-based suitability and climate change analysis, thereby addressing this concern and avoiding misinterpretation of the study’s scope.

*5) A significant weakness of the study is its failure to adequately incorporate and analyze crucial dengue epidemiological characteristics.*

Thank you for highlighting this point. We acknowledge that integrating additional dengue epidemiological characteristics can strengthen the depth of interpretation. However, the present study was designed specifically to examine spatial and temporal trends in relation to climatic heterogeneity at the national district level, using surveillance data that did not consistently include variables such as serotype distribution, age-specific incidence, or entomological indices.

However, a state-wise monthly climatology of dengue incidence for the period 2010-2022 is added and presented as a heatmap in the Supplementary Fig S1, which captures recurrent seasonal patterns and intra-annual variability across states.

**Reviewer #3:**

The study aims to explore the spatiotemporal distribution of dengue incidence across Indian districts, analyzing climatic influences using the Köppen-Geiger classification. It uses 13 years (2010-2022) district-level data, linear regression for temporal analysis, Kruskal-Wallis tests for inter-zonal incidence differences, and Moran’s I statistics for spatial autocorrelation and hotspot detection. Though the study addresses an important public health issue and has relevance but requires explanation to few concerns which are mentioned below.

*1. The manuscript does not clarify what new insights it offers beyond confirming known patterns of dengue expansion into semi-arid zones and the novelty of the study is limited, as several studies (e.g., Kakarla SG, et al., 2020; Hussain SS and Dhiman RC, 2022) have already explored dengue’s climatic determinants and spatial heterogeneity across India.*

Thank you for this important observation. While previous studies have examined climatic determinants and spatial heterogeneity of dengue in India, our study adds several novel insights that extend beyond confirming known patterns of dengue expansion into semi-arid regions.

1. ***Nationwide Köppen-Geiger Climate Zone Stratification***:

Unlike earlier studies that primarily focused on individual states/districts or broad climatic gradients, our analysis is the first to systematically quantify long-term dengue trends across all major Köppen-Geiger climate zones in India. This approach enables climate-specific trend estimation that has not been previously documented.

2. ***Longitudinal Trend Estimation Using Prais-Winsten Regression***:

We incorporate updated, zone-stratified Prais-Winsten regression models to account for serial autocorrelation, an analytical refinement not applied in earlier national-scale studies.

3. ***Comparative Assessment Across Climatically Distinct Zones***:

Our findings highlight unexpectedly steep increases in traditionally cooler zones (e.g., ET, Cfa, Dfb), providing new evidence of dengue amplification in climates historically considered low-risk, an insight not previously demonstrated in the existing literature.

Overall, these components provide a more climate zone-specific understanding of dengue dynamics across India, thereby extending the current evidence base.

*2. Line 53-55: Mention the recent statistics of dengue global burden (As per WHO, 14.6 million cases in 2024).*

Thank you for the suggestion. We have revised the manuscript to include the most recent global dengue statistics as reported by the WHO. The updated sentence now reads:

“According to the World Health Organization’s (WHO) global dengue surveillance data, 2024 recorded an unprecedented surge in dengue incidence, with more than 14.6 million reported cases and over 12,000 associated deaths across 100 countries.”

*3. Line 69-70: Total number of deaths from 2019-2024 is 1653 as per NCVBDC not 1592.*

Thank you for your careful review and for bringing this discrepancy to our attention. We have verified the data from the NCVBDC and corrected the total number of dengue-related deaths from 2019 to 2024 in the manuscript to 1,653.

*4. The previous spatial or climatic modelling studies were not mentioned in the introduction section which is important for positioning your work.*

Thank you for this valuable comment. We have now added a brief sentence in the Introduction to mention the previous spatial and climatic modelling studies on dengue.

“Several spatial and climatic modelling studies have examined the influence of environmental drivers on dengue transmission using approaches such as spatial autocorrelation (Mahato et al., 2025), spatial regression frameworks (e.g., Simultaneous autoregressive models [SAR], Conditional autoregressive [CAR], and Bayesian hierarchical models) (Aswi et al., 2018), ecological niche modelling (Ashby et al., 2017), and climate-disease time-series models (Alam et al., 2025)”.

*5. Since the present work highlights the dengue-climate suitability, I suggest to include a short explanatory line on how each climatic variable influence dengue transmission (e.g., temperature influences viral replication within the vector, while rainfall affects larval availability).*

Thank you for this valuable suggestion. We have revised the manuscript to include a concise explanation of how key climatic variables influence dengue transmission.

“Rising temperatures, increased rainfall, and prolonged humidity create favourable conditions for the proliferation of *Aedes* mosquitoes and the transmission of dengue (Abbasi, 2025a). Warmer climates accelerate viral incubation within mosquitoes, while moderate rainfall sustains breeding sites, and extreme weather events, such as floods and hurricanes, generate new ones (Alkhaldy, 2017; Kamiya et al., 2020). High humidity enhances mosquito survival and activity, whereas strong winds can limit dispersal and host contact (Gui et al., 2022). Overall, these climatic changes are expanding dengue into new regions and complicating control efforts.”

*6. Köppen-Geiger data period is from 1980-2016, however the dengue data extend to 2022. This temporal mismatch could bias interpretation. Climatic zones might have shifted over the study period due to warming trends.*

We appreciate the reviewer’s valuable observation regarding the temporal mismatch between the Köppen-Geiger climate classification data (1980-2016) and the dengue dataset (extending to 2022). However, the Köppen-Geiger classification is derived from multi-decadal averages and remains the most robust and globally consistent dataset for representing long-term climatic conditions. Its stability and widespread use in epidemiological and environmental research justify its application in this study. The climate zones may be affected by recent warming trends, which could introduce an insignificant degree of temporal bias. We have noted this as a methodological limitation in the revised manuscript.

*7. As annual dengue data may exhibit non-linearity and lag effects, time-series regression (e.g., distributed lag models) would be more appropriate than a simple linear regression model.*

We thank the reviewer for the helpful suggestion. We agree that distributed lag/time-series regression approaches are valuable for estimating short-term lagged effects and non-linear exposure-response relationships using high-resolution time series (e.g., daily or weekly data). However, our analysis uses annual, district-level dengue incidence (2010-2022). At this temporal resolution (13 annual observations per district), lagged and short-term effects cannot be meaningfully resolved.

We therefore used Prais-Winsten regression (PMID: 27869966, <https://cowles.yale.edu/sites/default/files/2023-05/s-0383.pdf>), which appropriately accounts for serial autocorrelation in time-dependent dengue incidence data (2010-2022). The analysis now estimates ‘Annual Increment Rates (AIR)’ with 95% confidence intervals and p-values to classify trends as increasing, decreasing, or stationary, following Antunes’ method.

We have added a limitation to the manuscript, noting that detection of short-term or lagged effects would require higher temporal resolution data (monthly/weekly).

*8. In the use of Moran’s I statistic, the authors did not indicate the conceptualization of spatial relation and the use of spatial weight matrix and how the distance threshold was calculated or chosen?*

Thank you for this valuable comment. In the revised manuscript, we have now detailed the construction of the spatial weight matrix. Specifically, we adopted a contiguity-based spatial conceptualization, using a first-order queen contiguity matrix, which considers districts sharing either a common boundary or a vertex as neighbours. This approach is widely used in district-level epidemiological studies because it captures both edge and corner adjacencies, providing a more realistic representation of spatial interaction for administrative units of varying shapes and sizes.

Corresponding methodological details have been added to the Methods-Spatial autocorrelation analysis section.

*9. Line 176: Table -1: The incidence rate denominator uses “2011-2022 projected census”. Mention the source and projection method for population data.*

Thank you for the comment. A brief explanation of the projection population data source and the projection method has been added to the Methods section (Subsection: Epidemiological and population data), with citations to the relevant sources.

*10. The discussion section looks repetitive and overly descriptive of the results rather than interpretive.*

We thank the reviewer for this constructive comment. In response, the Discussion section has been substantially revised to reduce repetition of the Results and to shift the emphasis from descriptive reporting to interpretation.

*11. Please ensure all abbreviations including Climate zone abbreviations (Aw, BWh, BSh) are defined at first mention for interdisciplinary readers.*

We have ensured that all abbreviations for the climate zones are provided in the Methodology (Subsection: Study Area and Climate Classification), where they are first mentioned.

*12. The expression of p-value “(p=Aw: 0.007; BSh: 0.003, BWh: 0.002)” is unclear. Mention the codes in the braces.*

Thank you for pointing this out. We have revised the sentence and added the corresponding Köppen-Geiger climate zone abbreviations for better clarity. The results presented now are based on the updated Prais-Winsten regression analysis.

*13. No mention of software used for spatial analysis, which is critical for reproducibility.*

We appreciate this important observation. In the revised manuscript, we have now stated that all spatial analyses and mapping were conducted using licensed ArcGIS (version 10.4), ensuring transparency and reproducibility of the methods.
