# Supplementary material for: Spatial distribution and risk assessment of dengue incidence at district level across major climatic zones in India
Source: PLoS One. 2026 Jun 9;21(6):e0350325. doi: 10.1371/journal.pone.0350325 (PMC13249156; doi:10.1371/journal.pone.0350325)
Supplement: Attachment — Submitted filename: Comments_Spatio-temporal pattern_Dengue_13.04.2026.docx [file pone.0350325.s006.docx]

**Comments on the manuscript entitled “** Spatial Distribution and Risk Assessment of Dengue Incidence at District Level Across Major Climatic Zones in India”

The study provides a comprehensive analysis of the spatial and temporal distribution of dengue incidence in various climatic zones of India using surveillance data at the district level from the National Programme for 13 years (2010-2022). The analysis is based on relatively updated data and analytical methods aiming to assess the significance of variability between the major climatic zones in dengue incidence and its clustering. The results are relevant to the public health programme as the information may be useful for planning - targeting and prioritising areas – for preventive measures.

However, there are a few minor corrections and clarifications that need to addressed, which are mentioned below.

1. Table. 1. Column 4: Please explain how these mean values for Annual Incidence Rate, Peak Annual Incidence Rate, Mean Annual Cases and Peak Annual Mean Cases were calculated so that it will be clear to the general readers.
2. Line 217 – 222: “Aw also contributed to **the largest proportion of the population in 2022 (32.57%) and mean annual cases (44,906.77),** suggesting a strong relationship between population density in tropical savanna regions and disease transmission. Similarly, climate zones such as Temperate dry winter (Cwa) and hot arid (BSh) reported high incidence rates and substantial case counts, with **Cwa covering the largest population proportion (36.91%) and reporting 22,188.54 mean annual cases”. –** this part of the para does not read well. It is not clear if the mean annual cases or incidence rates were compared with the proportion population. For a 32.57% population, the number of cases were 44,906 whereas for 36.91% population, the number of cases was only 22,188.54. Needs clarity.
3. Table 2.: Post-hoc Comparison (pair-wise Wilcoxon test), Z1/Z3: The P values for the years 2011 & 2012 indicate that there is no significant difference. But, it is shown that these are significant; Table - Foot Note: Significant at P>0.05 – to be corrected as P<0.05.
4. Line, 236 -238: Though the AIR indicated a significant upward trend in these zones, there is a wide variability as indicated by 95% CI. This is to be mentioned and discussed.

The manuscript needs minor revision.

*********
