# Supplementary material for: Spatial distribution and risk assessment of dengue incidence at district level across major climatic zones in India
Source: PLoS One. 2026 Jun 9;21(6):e0350325. doi: 10.1371/journal.pone.0350325 (PMC13249156; doi:10.1371/journal.pone.0350325)
Supplement: Attachment — Submitted filename: Response to Reviewers.docx [file pone.0350325.s007.docx]

**Authors Response to Reviewers**

**Revision no. 2**

**Manuscript Title:** Spatial Distribution and Risk Assessment of Dengue Incidence at District Level Across Major Climatic Zones in India

**Manuscript No.** PONE-D-25-42541R2

**Reviewer #2:**

*Thank you so much for addressing all comments properly. I understood your concern about your objectives.*

We sincerely thank the reviewer for the positive feedback and for taking the time to carefully evaluate our manuscript. We are glad that the revisions adequately addressed the concerns raised.

**Reviewer #4:**

*While a statement has been provided in the submission system, it is not included within the main manuscript text. Please add a dedicated “Data Availability” section in the manuscript to comply with journal requirements.*

We thank the reviewer for this observation. As per the journal’s procedure, the data availability statement was previously provided in the submission system. The statement reads: "All relevant data are within the manuscript and its Supporting Information files," which will be typeset upon acceptance of the manuscript.

**Reviewer #5:**

*The authors have adequately addressed the previous reviewers' comments and present a clear, scientific study of the impact of climate on dengue incidence in India.*

We sincerely thank the reviewer for the positive and encouraging feedback, and for recognizing the scientific merit of our study. We are glad that the revisions met the reviewer's expectations and that the manuscript's objectives and findings are clearly presented.

**Reviewer #6:**

*The study provides a comprehensive analysis of the spatial and temporal distribution of dengue incidence in various climatic zones of India using surveillance data at the district level from the National Programme for 13 years (2010-2022). The analysis is based on relatively updated data and analytical methods aiming to assess the significance of variability between the major climatic zones in dengue incidence and its clustering. The results are relevant to the public health programme as the information may be useful for planning - targeting and prioritising areas – for preventive measures.*

We sincerely thank the reviewer for the thorough review of our manuscript. We are pleased that the reviewer recognized the comprehensiveness of our analysis, the relevance of the 13-year district-level surveillance data, and the public health significance of our findings.

*However, there are a few minor corrections and clarifications that need to addressed, which are mentioned below.*

1. *Table. 1. Column 4: Please explain how these mean values for Annual Incidence Rate, Peak Annual Incidence Rate, Mean Annual Cases and Peak Annual Mean Cases were calculated so that it will be clear to the general readers.*

We thank the reviewer for this helpful suggestion. We have added a footnote to Table 1 (Line 222-229) to clarify the calculation method for all four metrics, as described below:

**Mean Annual Incidence Rate (per 100,000 people):** Annual incidence rates were calculated for each district as ([cases ÷ projected population] × 100,000) and averaged across all 13 study years (2010-2022) and districts within each climate zone.

**Peak Mean Annual Incidence Rate (per 100,000 people):** The three highest annual incidence rates during 2010-2022 were identified for each climate zone and averaged. **Mean Annual Cases:** The total number of cases across all districts within each climate zone was summed for each year, and then averaged over the study years.

**Peak Annual Mean Cases:** The total number of cases across all districts within each climate zone was summed for each year, and then averaged across the three peak years.

Also, we have standardized all numerical values in Table 1 to two decimal places for consistency and uniformity across the table.

2. *Line 217 – 222: “Aw also contributed to* ***the largest proportion of the population in 2022 (32.57%) and mean annual cases (44,906.77),*** *suggesting a strong relationship between population density in tropical savanna regions and disease transmission. Similarly, climate zones such as Temperate dry winter (Cwa) and hot arid (BSh) reported high incidence rates and substantial case counts, with* ***Cwa covering the largest population proportion (36.91%) and reporting 22,188.54 mean annual cases”. –*** *this part of the para does not read well. It is not clear if the mean annual cases or incidence rates were compared with the proportion population. For a 32.57% population, the number of cases were 44,906 whereas for 36.91% population, the number of cases was only 22,188.54. Needs clarity.*

We thank the reviewer for this helpful observation. We have revised the paragraph for better clarity (line no. 201-207).

3. *Table 2.: Post-hoc Comparison (pair-wise Wilcoxon test), Z1/Z3: The P values for the years 2011 & 2012 indicate that there is no significant difference. But, it is shown that these are significant; Table – Foot Note: Significant at P>0.05 – to be corrected as P<0.05.*

We sincerely apologize for this typographical error in the table footnote. This has been corrected in the revised manuscript. We confirm that the p-values for the years 2011 and 2012 in the Z1/Z3 comparison do not reach statistical significance at P<0.05, and the significance markers for these years have been removed accordingly.

4. *Line, 236 -238: Though the AIR indicated a significant upward trend in these zones, there is a wide variability as indicated by 95% CI. This is to be mentioned and discussed.*

We thank the reviewer for this important observation. We agree that while the AIR indicated statistically significant upward trends in ET, Cfa, and Dfb, the wide 95% confidence intervals reflect considerable variability in these estimates. We have revised the text (line no. 247-255) to acknowledge this variability, attributing it to the small number of districts, low mean annual case counts, and negligible population proportions in these zones, which render the trend estimates sensitive to minor fluctuations in case numbers.
