# Supplementary material for: Spatial distribution and risk assessment of dengue incidence at district level across major climatic zones in India
Source: PLoS One. 2026 Jun 9;21(6):e0350325. doi: 10.1371/journal.pone.0350325 (PMC13249156; doi:10.1371/journal.pone.0350325)
Supplement: Peer review history

# Peer review: Decision Letter 0

**DOI:** 10.1371/journal.pone.0350325.r001

**Authors:** Manob Das

5 Nov 2025

PONE-D-25-42541

Spatial Distribution and Risk Assessment of Dengue Incidence at District Level Across Major Climatic Zones in India

PLOS ONE

Dear Dr. Behera,

Thank you for submitting your manuscript to PLOS ONE. After careful consideration, we feel that it has merit but does not fully meet PLOS ONE’s publication criteria as it currently stands. Therefore, we invite you to submit a revised version of the manuscript that addresses the points raised during the review process.

Please submit your revised manuscript by Dec 20 2025 11:59PM. If you will need more time than this to complete your revisions, please reply to this message or contact the journal office at plosone@plos.org. When you're ready to submit your revision, log on to https://www.editorialmanager.com/pone/ and select the 'Submissions Needing Revision' folder to locate your manuscript file.

Please include the following items when submitting your revised manuscript:

- A rebuttal letter that responds to each point raised by the academic editor and reviewer(s). You should upload this letter as a separate file labeled 'Response to Reviewers'.
- A marked-up copy of your manuscript that highlights changes made to the original version. You should upload this as a separate file labeled 'Revised Manuscript with Track Changes'.
- An unmarked version of your revised paper without tracked changes. You should upload this as a separate file labeled 'Manuscript'.

If you would like to make changes to your financial disclosure, please include your updated statement in your cover letter. Guidelines for resubmitting your figure files are available below the reviewer comments at the end of this letter.

If applicable, we recommend that you deposit your laboratory protocols in protocols.io to enhance the reproducibility of your results. Protocols.io assigns your protocol its own identifier (DOI) so that it can be cited independently in the future. For instructions see: https://journals.plos.org/plosone/s/submission-guidelines#loc-laboratory-protocols. Additionally, PLOS ONE offers an option for publishing peer-reviewed Lab Protocol articles, which describe protocols hosted on protocols.io. Read more information on sharing protocols at https://plos.org/protocols?utm_medium=editorial-email&utm_source=authorletters&utm_campaign=protocols.

We look forward to receiving your revised manuscript.

Kind regards,

Dr. Manob Das

Academic Editor

PLOS ONE

Journal Requirements:

When submitting your revision, we need you to address these additional requirements.

1. Please ensure that your manuscript meets PLOS ONE's style requirements, including those for file naming. The PLOS ONE style templates can be found at

https://journals.plos.org/plosone/s/file?id=wjVg/PLOSOne_formatting_sample_main_body.pdf and

https://journals.plos.org/plosone/s/file?id=ba62/PLOSOne_formatting_sample_title_authors_affiliations.pdf

2. Thank you for stating the following in the Acknowledgments Section of your manuscript:

“The first author, Meenu Mariya James, gratefully acknowledges the Indian Council of Social Science Research (ICSSR) for the award of a doctoral fellowship, which provided valuable support  for this research.”

We note that you have provided funding information that is not currently declared in your Funding Statement. However, funding information should not appear in the Acknowledgments section or other areas of your manuscript. We will only publish funding information present in the Funding Statement section of the online submission form.

Please remove any funding-related text from the manuscript and let us know how you would like to update your Funding Statement. Currently, your Funding Statement reads as follows:

“This research was supported by the Indian Council of Social Science Research (ICSSR) through the award of a doctoral fellowship to the first author, Meenu Mariya James (Award Letter No. ICSSR/RFD/24-25/ENV/GEN/68) (https://icssr.org/doctoral-fellowship).”

Please include your amended statements within your cover letter; we will change the online submission form on your behalf.

3. Thank you for stating the following financial disclosure:

“This research was supported by the Indian Council of Social Science Research (ICSSR) through the award of a doctoral fellowship to the first author, Meenu Mariya James (Award Letter No. ICSSR/RFD/24-25/ENV/GEN/68) (https://icssr.org/doctoral-fellowship).”

Please state what role the funders took in the study.  If the funders had no role, please state: "The funders had no role in study design, data collection and analysis, decision to publish, or preparation of the manuscript."

If this statement is not correct you must amend it as needed.

Please include this amended Role of Funder statement in your cover letter; we will change the online submission form on your behalf.

4. PLOS requires an ORCID iD for the corresponding author in Editorial Manager on papers submitted after December 6th, 2016. Please ensure that you have an ORCID iD and that it is validated in Editorial Manager. To do this, go to ‘Update my Information’ (in the upper left-hand corner of the main menu), and click on the Fetch/Validate link next to the ORCID field. This will take you to the ORCID site and allow you to create a new iD or authenticate a pre-existing iD in Editorial Manager.

5. We note that Figure 1 and 3 in your submission contain map images which may be copyrighted. All PLOS content is published under the Creative Commons Attribution License (CC BY 4.0), which means that the manuscript, images, and Supporting Information files will be freely available online, and any third party is permitted to access, download, copy, distribute, and use these materials in any way, even commercially, with proper attribution. For these reasons, we cannot publish previously copyrighted maps or satellite images created using proprietary data, such as Google software (Google Maps, Street View, and Earth). For more information, see our copyright guidelines:

http://journals.plos.org/plosone/s/licenses-and-copyright.

We require you to either (1) present written permission from the copyright holder to publish these figures specifically under the CC BY 4.0 license, or (2) remove the figures from your submission:

1. You may seek permission from the original copyright holder of Figure(s) [#] to publish the content specifically under the CC BY 4.0 license.

We recommend that you contact the original copyright holder with the Content Permission Form (http://journals.plos.org/plosone/s/file?id=7c09/content-permission-form.pdf) and the following text:

“I request permission for the open-access journal PLOS ONE to publish XXX under the Creative Commons Attribution License (CCAL) CC BY 4.0 (http://creativecommons.org/licenses/by/4.0/). Please be aware that this license allows unrestricted use and distribution, even commercially, by third parties. Please reply and provide explicit written permission to publish XXX under a CC BY license and complete the attached form.”

Please upload the completed Content Permission Form or other proof of granted permissions as an "Other" file with your submission.

In the figure caption of the copyrighted figure, please include the following text: “Reprinted from [ref] under a CC BY license, with permission from [name of publisher], original copyright [original copyright year].”

2. If you are unable to obtain permission from the original copyright holder to publish these figures under the CC BY 4.0 license or if the copyright holder’s requirements are incompatible with the CC BY 4.0 license, please either i) remove the figure or ii) supply a replacement figure that complies with the CC BY 4.0 license. Please check copyright information on all replacement figures and update the figure caption with source information. If applicable, please specify in the figure caption text when a figure is similar but not identical to the original image and is therefore for illustrative purposes only.

The following resources for replacing copyrighted map figures may be helpful:

USGS National Map Viewer (public domain): http://viewer.nationalmap.gov/viewer/

The Gateway to Astronaut Photography of Earth (public domain): http://eol.jsc.nasa.gov/sseop/clickmap/

Maps at the CIA (public domain): https://www.cia.gov/library/publications/the-world-factbook/index.html and https://www.cia.gov/library/publications/cia-maps-publications/index.html

NASA Earth Observatory (public domain): http://earthobservatory.nasa.gov/

Landsat: http://landsat.visibleearth.nasa.gov/

USGS EROS (Earth Resources Observatory and Science (EROS) Center) (public domain): http://eros.usgs.gov/#

Natural Earth (public domain): http://www.naturalearthdata.com/

6. If the reviewer comments include a recommendation to cite specific previously published works, please review and evaluate these publications to determine whether they are relevant and should be cited. There is no requirement to cite these works unless the editor has indicated otherwise.

[Note: HTML markup is below. Please do not edit.]

Reviewers' comments:

Reviewer's Responses to Questions

**Comments to the Author**

1. Is the manuscript technically sound, and do the data support the conclusions?

The manuscript must describe a technically sound piece of scientific research with data that supports the conclusions. Experiments must have been conducted rigorously, with appropriate controls, replication, and sample sizes. The conclusions must be drawn appropriately based on the data presented.

Reviewer #1: Yes

Reviewer #2: Yes

Reviewer #3: Partly

**********

2. Has the statistical analysis been performed appropriately and rigorously?

Reviewer #1: Yes

Reviewer #2: No

Reviewer #3: Yes

**********

3. Have the authors made all data underlying the findings in their manuscript fully available?

The PLOS Data policy requires authors to make all data underlying the findings described in their manuscript fully available without restriction, with rare exception (please refer to the Data Availability Statement in the manuscript PDF file). The data should be provided as part of the manuscript or its supporting information, or deposited to a public repository. For example, in addition to summary statistics, the data points behind means, medians and variance measures should be available. If there are restrictions on publicly sharing data—e.g. participant privacy or use of data from a third party—those must be specified.

Reviewer #1: No

Reviewer #2: Yes

Reviewer #3: No

**********

4. Is the manuscript presented in an intelligible fashion and written in standard English?

PLOS ONE does not copyedit accepted manuscripts, so the language in submitted articles must be clear, correct, and unambiguous. Any typographical or grammatical errors should be corrected at revision, so please note any specific errors here.

Reviewer #1: Yes

Reviewer #2: Yes

Reviewer #3: Yes

**********

5. Review Comments to the Author

Please use the space provided to explain your answers to the questions above. You may also include additional comments for the author, including concerns about dual publication, research ethics, or publication ethics. (Please upload your review as an attachment if it exceeds 20,000 characters)

Reviewer #1: This study is important and epidemiologically useful as it provides a comprehensive analysis of the spatiotemporal dynamics of dengue incidence in various climatic zones from India. The analysis that shows how climate and demographic variability has affected understanding the spatial patterns of its incidence and transmission is also important. These two aspects are of clinical and preventive value apply to other contexts.

The authors need to make all the data underlying the findings in their manuscript fully available upon request by the journal.

Reviewer #2: I have gone the manuscript thoroughly, and my review feedback as followed.

1) The study based on retrospective dengue data spanning from 2010 to 2022. However, the authors' used linear regression for temporal trend analysis is a significant methodological flaw, as it fails to account for the serial correlation inherent in time-dependent data. A time series analysis would have been the correct approach.

2) The study has a potential for bias because it did not account for the fact that the locations where infections were acquired and where patients received treatment were often different. This discrepancy could lead to an inaccurate representation of dengue incidence in specific areas.

3) Conclusion in abstract is very short and understand to relate their results.

4) A more appropriate approach would have been to analyze weather variability instead of climate change variability, given that the study used only 12 years of data. Climate change trends are typically assessed over much longer periods, such as 30 years or more.

5) A significant weakness of the study is its failure to adequately incorporate and analyze crucial dengue epidemiological characteristics.

Reviewer #3: The study aims to explore the spatiotemporal distribution of dengue incidence across Indian districts, analyzing climatic influences using the Köppen-Geiger classification. It uses 13 years (2010-2022) district-level data, linear regression for temporal analysis, Kruskal-Wallis tests for inter-zonal incidence differences, and Moran’s I statistics for spatial autocorrelation and hotspot detection. Though the study addresses an important public health issue and has relevance but requires explanation to few concerns which are mentioned below.

1. The manuscript does not clarify what new insights it offers beyond confirming known patterns of dengue expansion into semi-arid zones and the novelty of the study is limited, as several studies (e.g., Kakarla SG, et al., 2020; Hussain SS and Dhiman RC, 2022) have already explored dengue’s climatic determinants and spatial heterogeneity across India.

2. Line 53-55: Mention the recent statistics of dengue global burden (As per WHO, 14.6 million cases in 2024).

3. Line 69-70: Total number of deaths from 2019-2024 is 1653 as per NCVBDC not 1592.

4. The previous spatial or climatic modelling studies were not mentioned in the introduction section which is important for positioning your work.

5. Since the present work highlights the dengue-climate suitability, I suggest to include a short explanatory line on how each climatic variable influence dengue transmission (e.g., temperature influences viral replication within the vector, while rainfall affects larval availability).

6. Köppen-Geiger data period is from 1980-2016, however the dengue data extend to 2022. This temporal mismatch could bias interpretation. Climatic zones might have shifted over the study period due to warming trends.

7. As annual dengue data may exhibit non linearity and lag effects, time-series regression (e.g., distributed lag models) would be more appropriate than simple linear regression model.

8. In the use of Moran’s I statistic, the authors did not indicate the conceptualization of spatial relation and the use of spatial weight matrix and how the distance threshold was calculated or chosen?

9. Line 176: Table -1: The incidence rate denominator uses “2011-2022 projected census”. Mention the source and projection method for population data.

10. The discussion section looks repetitive and overly descriptive of the results rather than interpretive.

11. Please ensure all abbreviations including Climate zone abbreviations (Aw, BWh, BSh) are defined at first mention for interdisciplinary readers.

12. The expression of p-value “(p=Aw: 0.007; BSh: 0.003, BWh: 0.002)” is unclear. Mention the codes in the braces.

13. No mention of software used for spatial analysis which is critical for reproducibility.

**********

6. PLOS authors have the option to publish the peer review history of their article (what does this mean?). If published, this will include your full peer review and any attached files.

If you choose “no”, your identity will remain anonymous but your review may still be made public.

**Do you want your identity to be public for this peer review?** For information about this choice, including consent withdrawal, please see our Privacy Policy.

Reviewer #1: No

Reviewer #2: No

Reviewer #3: No

**********

[NOTE: If reviewer comments were submitted as an attachment file, they will be attached to this email and accessible via the submission site. Please log into your account, locate the manuscript record, and check for the action link "View Attachments". If this link does not appear, there are no attachment files.]

To ensure your figures meet our technical requirements, please review our figure guidelines: https://journals.plos.org/plosone/s/figures

You may also use PLOS’s free figure tool, NAAS, to help you prepare publication quality figures: https://journals.plos.org/plosone/s/figures#loc-tools-for-figure-preparation.

NAAS will assess whether your figures meet our technical requirements by comparing each figure against our figure specifications.

# Author comment: Author response to Decision Letter 1

**DOI:** 10.1371/journal.pone.0350325.r002

31 Dec 2025

We thank the Editor and the reviewers for their thorough evaluation of our manuscript and for their constructive and insightful comments. We have carefully considered all suggestions and have revised the manuscript extensively to address methodological, conceptual, and presentation-related concerns. Key revisions include the refinement of temporal trend analysis using Prais-Winsten regression to account for serial autocorrelation, clearer presentation of the study’s novelty and contribution within the existing literature, improved justification and explanation of the climatic framework, and a strengthened discussion section. We believe that these revisions have substantially improved the scientific rigour, clarity, and interpretability of the manuscript. We provide detailed point-by-point responses below.

Reviewer #1:

This study is important and epidemiologically useful as it provides a comprehensive analysis of the spatiotemporal dynamics of dengue incidence in various climatic zones from India. The analysis that shows how climate and demographic variability has affected understanding the spatial patterns of its incidence and transmission is also important. These two aspects are of clinical and preventive value apply to other contexts.

The authors need to make all the data underlying the findings in their manuscript fully available upon request by the journal.

We thank the reviewer for their careful review of our manuscript. All data underlying the findings reported in this study are included directly within the manuscript or provided in the supplementary information. Additional clarification or supporting materials will be supplied upon request by the journal.

Reviewer #2:

I have gone the manuscript thoroughly, and my review feedback as followed.

1) The study based on retrospective dengue data spanning from 2010 to 2022. However, the authors' used linear regression for temporal trend analysis is a significant methodological flaw, as it fails to account for the serial correlation inherent in time-dependent data. A time series analysis would have been the correct approach.

Thank you for highlighting the methodological concern regarding the use of linear regression for temporal trend analysis. We agree that distributed time-series analysis approaches are valuable for estimating short-term lagged effects and non-linear exposure-response relationships using high-resolution time series (e.g., daily or weekly data). However, our analysis uses annual, district-level dengue incidence (2010-2022). At this temporal resolution (13 annual observations per district), lagged and short-term effects cannot be meaningfully resolved using time-series analysis.

In response, we have revised our approach and now employ Prais-Winsten regression (PMID: 27869966, https://cowles.yale.edu/sites/default/files/2023-05/s-0383.pdf), which appropriately accounts for serial autocorrelation in time-dependent dengue incidence data (2010-2022). The analysis now estimates ‘Annual Increment Rates (AIR)’ with 95% confidence intervals and p-values to classify trends as increasing, decreasing, or stationary, following Antunes’ method.

All changes have been updated in the manuscript (Methodology: Temporal Trend Analysis).

2) The study has a potential for bias because it did not account for the fact that the locations where infections were acquired and where patients received treatment were often different. This discrepancy could lead to an inaccurate representation of dengue incidence in specific areas.

We agree with the reviewer that discrepancies between infection acquisition sites and treatment locations may introduce spatial misclassification. This is an inherent limitation of surveillance-based dengue datasets. At the same time, we note that this bias is likely limited because the administrative units used (districts) are geographically large, making it more probable that infection acquisition and treatment occur within the same district. However, we have now explicitly acknowledged this potential bias in the Limitations section and clarified that reported case locations may not fully represent the true spatial distribution of transmission.

3) Conclusion in abstract is very short and understand to relate their results.

Thank you for this observation. We have revised the concluding statement of the abstract to more clearly reflect and integrate our key temporal and spatial findings.

4) A more appropriate approach would have been to analyze weather variability instead of climate change variability, given that the study used only 12 years of data. Climate change trends are typically assessed over much longer periods, such as 30 years or more.

Thank you for this important observation. We would like to clarify that the primary focus of this study is climatic zonation, rather than assessing climate change trends. The Köppen-Geiger climate classification used in this study is derived from long-term climatological normals (1980-2016) (PMID: 30375988) and is intended to represent relatively stable, multi-decadal climatic regimes rather than short-term weather variability or recent climate change signals.

Our analysis examines how dengue incidence patterns vary across these established climate zones, using surveillance data from 2010 to 2022, without attempting to infer trends related to climate change. We have revised the manuscript to more explicitly distinguish between climate zone-based suitability and climate change analysis, thereby addressing this concern and avoiding misinterpretation of the study’s scope.

5) A significant weakness of the study is its failure to adequately incorporate and analyze crucial dengue epidemiological characteristics.

Thank you for highlighting this point. We acknowledge that integrating additional dengue epidemiological characteristics can strengthen the depth of interpretation. However, the present study was designed specifically to examine spatial and temporal trends in relation to climatic heterogeneity at the national district level, using surveillance data that did not consistently include variables such as serotype distribution, age-specific incidence, or entomological indices.

However, a state-wise monthly climatology of dengue incidence for the period 2010-2022 is added and presented as a heatmap in the Supplementary Fig S1, which captures recurrent seasonal patterns and intra-annual variability across states.

Reviewer #3:

The study aims to explore the spatiotemporal distribution of dengue incidence across Indian districts, analyzing climatic influences using the Köppen-Geiger classification. It uses 13 years (2010-2022) district-level data, linear regression for temporal analysis, Kruskal-Wallis tests for inter-zonal incidence differences, and Moran’s I statistics for spatial autocorrelation and hotspot detection. Though the study addresses an important public health issue and has relevance but requires explanation to few concerns which are mentioned below.

1. The manuscript does not clarify what new insights it offers beyond confirming known patterns of dengue expansion into semi-arid zones and the novelty of the study is limited, as several studies (e.g., Kakarla SG, et al., 2020; Hussain SS and Dhiman RC, 2022) have already explored dengue’s climatic determinants and spatial heterogeneity across India.

Thank you for this important observation. While previous studies have examined climatic determinants and spatial heterogeneity of dengue in India, our study adds several novel insights that extend beyond confirming known patterns of dengue expansion into semi-arid regions.

1. Nationwide Köppen-Geiger Climate Zone Stratification:

Unlike earlier studies that primarily focused on individual states/districts or broad climatic gradients, our analysis is the first to systematically quantify long-term dengue trends across all major Köppen-Geiger climate zones in India. This approach enables climate-specific trend estimation that has not been previously documented.

2. Longitudinal Trend Estimation Using Prais-Winsten Regression:

We incorporate updated, zone-stratified Prais-Winsten regression models to account for serial autocorrelation, an analytical refinement not applied in earlier national-scale studies.

3. Comparative Assessment Across Climatically Distinct Zones:

Our findings highlight unexpectedly steep increases in traditionally cooler zones (e.g., ET, Cfa, Dfb), providing new evidence of dengue amplification in climates historically considered low-risk, an insight not previously demonstrated in the existing literature.

Overall, these components provide a more climate zone-specific understanding of dengue dynamics across India, thereby extending the current evidence base.

2. Line 53-55: Mention the recent statistics of dengue global burden (As per WHO, 14.6 million cases in 2024).

Thank you for the suggestion. We have revised the manuscript to include the most recent global dengue statistics as reported by the WHO. The updated sentence now reads:

“According to the World Health Organization’s (WHO) global dengue surveillance data, 2024 recorded an unprecedented surge in dengue incidence, with more than 14.6 million reported cases and over 12,000 associated deaths across 100 countries.”

3. Line 69-70: Total number of deaths from 2019-2024 is 1653 as per NCVBDC not 1592.

Thank you for your careful review and for bringing this discrepancy to our attention. We have verified the data from the NCVBDC and corrected the total number of dengue-related deaths from 2019 to 2024 in the manuscript to 1,653.

4. The previous spatial or climatic modelling studies were not mentioned in the introduction section which is important for positioning your work.

Thank you for this valuable comment. We have now added a brief sentence in the Introduction to mention the previous spatial and climatic modelling studies on dengue.

“Several spatial and climatic modelling studies have examined the influence of environmental drivers on dengue transmission using approaches such as spatial autocorrelation (Mahato et al., 2025), spatial regression frameworks (e.g., Simultaneous autoregressive models [SAR], Conditional autoregressive [CAR], and Bayesian hierarchical models) (Aswi et al., 2018), ecological niche modelling (Ashby et al., 2017), and climate-disease time-series models (Alam et al., 2025)”.

5. Since the present work highlights the dengue-climate suitability, I suggest to include a short explanatory line on how each climatic variable influence dengue transmission (e.g., temperature influences viral replication within the vector, while rainfall affects larval availability).

Thank you for this valuable suggestion. We have revised the manuscript to include a concise explanation of how key climatic variables influence dengue transmission.

“Rising temperatures, increased rainfall, and prolonged humidity create favourable conditions for the proliferation of Aedes mosquitoes and the transmission of dengue (Abbasi, 2025a). Warmer climates accelerate viral incubation within mosquitoes, while moderate rainfall sustains breeding sites, and extreme weather events, such as floods and hurricanes, generate new ones (Alkhaldy, 2017; Kamiya et al., 2020). High humidity enhances mosquito survival and activity, whereas strong winds can limit dispersal and host contact (Gui et al., 2022). Overall, these climatic changes are expanding dengue into new regions and complicating control efforts.”

6. Köppen-Geiger data period is from 1980-2016, however the dengue data extend to 2022. This temporal mismatch could bias interpretation. Climatic zones might have shifted over the study period due to warming trends.

We appreciate the reviewer’s valuable observation regarding the temporal mismatch between the Köppen-Geiger climate classification data (1980-2016) and the dengue dataset (extending to 2022). However, the Köppen-Geiger classification is derived from multi-decadal averages and remains the most robust and globally consistent dataset for representing long-term climatic conditions. Its stability and widespread use in epidemiological and environmental research justify its application in this study. The climate zones may be affected by recent warming trends, which could introduce an insignificant degree of temporal bias. We have noted this as a methodological limitation in the revised manuscript.

7. As annual dengue data may exhibit non-linearity and lag effects, time-series regression (e.g., distributed lag models) would be more appropriate than a simple linear regression model.

We thank the reviewer for the helpful suggestion. We agree that distributed lag/time-series regression approaches are valuable for estimating short-term lagged effects and non-linear exposure-response relationships using high-resolution time series (e.g., daily or weekly data). However, our analysis uses annual, district-level dengue incidence (2010-2022). At this temporal resolution (13 annual observations per district), lagged and short-term effects cannot be meaningfully resolved.

We therefore used Prais-Winsten regression (PMID: 27869966, https://cowles.yale.edu/sites/default/files/2023-05/s-0383.pdf), which appropriately accounts for serial autocorrelation in time-dependent dengue incidence data (2010-2022). The analysis now estimates ‘Annual Increment Rates (AIR)’ with 95% confidence intervals and p-values to classify trends as increasing, decreasing, or stationary, following Antunes’ method.

We have added a limitation to the manuscript, noting that detection of short-term or lagged effects would require higher temporal resolution data (monthly/weekly).

8. In the use of Moran’s I statistic, the authors did not indicate the conceptualization of spatial relation and the use of spatial weight matrix and how the distance threshold was calculated or chosen?

Thank you for this valuable comment. In the revised manuscript, we have now detailed the construction of the spatial weight matrix. Specifically, we adopted a contiguity-based spatial conceptualization, using a first-order queen contiguity matrix, which considers districts sharing either a common boundary or a vertex as neighbours. This approach is widely used in district-level epidemiological studies because it captures both edge and corner adjacencies, providing a more realistic representation of spatial interaction for administrative units of varying shapes and sizes.

Corresponding methodological details have been added to the Methods-Spatial autocorrelation analysis section.

9. Line 176: Table -1: The incidence rate denominator uses “2011-2022 projected census”. Mention the source and projection method for population data.

Thank you for the comment. A brief explanation of the projection population data source and the projection method has been added to the Methods section (Subsection: Epidemiological and population data), with citations to the relevant sources.

10. The discussion section looks repetitive and overly descriptive of the results rather than interpretive.

We thank the reviewer for this constructive comment. In response, the Discussion section has been substantially revised to reduce repetition of the Results and to shift the emphasis from descriptive reporting to interpretation.

11. Please ensure all abbreviations including Climate zone abbreviations (Aw, BWh, BSh) are defined at first mention for interdisciplinary readers.

We have ensured that all abbreviations for the climate zones are provided in the Methodology (Subsection: Study Area and Climate Classification), where they are first mentioned.

12. The expression of p-value “(p=Aw: 0.007; BSh: 0.003, BWh: 0.002)” is unclear. Mention the codes in the braces.

Thank you for pointing this out. We have revised the sentence and added the corresponding Köppen-Geiger climate zone abbreviations for better clarity. The results presented now are based on the updated Prais-Winsten regression analysis.

13. No mention of software used for spatial analysis, which is critical for reproducibility.

We appreciate this important observation. In the revised manuscript, we have now stated that all spatial analyses and mapping were conducted using licensed ArcGIS (version 10.4), ensuring transparen

Attachment Submitted filename: Authors Response Sheet.docx

# Peer review: Decision Letter 1

**DOI:** 10.1371/journal.pone.0350325.r003

**Authors:** Rajib Chowdhury

15 Apr 2026

PONE-D-25-42541R1

Spatial Distribution and Risk Assessment of Dengue Incidence at District Level Across Major Climatic Zones in India

PLOS One

Dear Dr. Behera,

Thank you for submitting your manuscript to PLOS ONE. After careful consideration, we feel that it has merit but does not fully meet PLOS ONE’s publication criteria as it currently stands. Therefore, we invite you to submit a revised version of the manuscript that addresses the points raised during the review process.

Please submit your revised manuscript by May 30 2026 11:59PM. If you will need more time than this to complete your revisions, please reply to this message or contact the journal office at plosone@plos.org. When you're ready to submit your revision, log on to https://www.editorialmanager.com/pone/ and select the 'Submissions Needing Revision' folder to locate your manuscript file.

Please include the following items when submitting your revised manuscript:

- A letter that responds to each point raised by the academic editor and reviewer(s). You should upload this letter as a separate file labeled 'Response to Reviewers'.
- A marked-up copy of your manuscript that highlights changes made to the original version. You should upload this as a separate file labeled 'Revised Manuscript with Track Changes'.
- An unmarked version of your revised paper without tracked changes. You should upload this as a separate file labeled 'Manuscript'.

If you would like to make changes to your financial disclosure, please include your updated statement in your cover letter. Guidelines for resubmitting your figure files are available below the reviewer comments at the end of this letter.

If applicable, we recommend that you deposit your laboratory protocols in protocols.io to enhance the reproducibility of your results. Protocols.io assigns your protocol its own identifier (DOI) so that it can be cited independently in the future. For instructions see: https://journals.plos.org/plosone/s/submission-guidelines#loc-laboratory-protocols. Additionally, PLOS ONE offers an option for publishing peer-reviewed Lab Protocol articles, which describe protocols hosted on protocols.io. Read more information on sharing protocols at https://plos.org/protocols?utm_medium=editorial-email&utm_source=authorletters&utm_campaign=protocols.

As the corresponding author, your ORCID iD is verified in the submission system and will appear in the published article. PLOS supports the use of ORCID, and we encourage all coauthors to register for an ORCID iD and use it as well. Please encourage your coauthors to verify their ORCID iD within the submission system before final acceptance, as unverified ORCID iDs will not appear in the published article. *Only* the individual author can complete the verification step; PLOS staff *cannot* verify ORCID iDs on behalf of authors.

We look forward to receiving your revised manuscript.

Kind regards,

Rajib Chowdhury, M.Sc.; MPH

Academic Editor

PLOS One

Journal Requirements:

1. If the reviewer comments include a recommendation to cite specific previously published works, please review and evaluate these publications to determine whether they are relevant and should be cited. There is no requirement to cite these works unless the editor has indicated otherwise.

2. Please review your reference list to ensure that it is complete and correct. If you have cited papers that have been retracted, please include the rationale for doing so in the manuscript text, or remove these references and replace them with relevant current references. Any changes to the reference list should be mentioned in the rebuttal letter that accompanies your revised manuscript. If you need to cite a retracted article, indicate the article’s retracted status in the References list and also include a citation and full reference for the retraction notice.

[Note: HTML markup is below. Please do not edit.]

Reviewer's Responses to Questions

**Comments to the Author**

1. If the authors have adequately addressed your comments raised in a previous round of review and you feel that this manuscript is now acceptable for publication, you may indicate that here to bypass the “Comments to the Author” section, enter your conflict of interest statement in the “Confidential to Editor” section, and submit your "Accept" recommendation.

Reviewer #2: All comments have been addressed

Reviewer #4: All comments have been addressed

Reviewer #5: All comments have been addressed

Reviewer #6: (No Response)

**********

2. Is the manuscript technically sound, and do the data support the conclusions?

The manuscript must describe a technically sound piece of scientific research with data that supports the conclusions. Experiments must have been conducted rigorously, with appropriate controls, replication, and sample sizes. The conclusions must be drawn appropriately based on the data presented.

Reviewer #2: Yes

Reviewer #4: Partly

Reviewer #5: Yes

Reviewer #6: Yes

**********

3. Has the statistical analysis been performed appropriately and rigorously?

Reviewer #2: Yes

Reviewer #4: Yes

Reviewer #5: Yes

Reviewer #6: Yes

**********

4. Have the authors made all data underlying the findings in their manuscript fully available?

The PLOS Data policy requires authors to make all data underlying the findings described in their manuscript fully available without restriction, with rare exception (please refer to the Data Availability Statement in the manuscript PDF file). The data should be provided as part of the manuscript or its supporting information, or deposited to a public repository. For example, in addition to summary statistics, the data points behind means, medians and variance measures should be available. If there are restrictions on publicly sharing data—e.g. participant privacy or use of data from a third party—those must be specified.

Reviewer #2: Yes

Reviewer #4: No

Reviewer #5: Yes

Reviewer #6: Yes

**********

5. Is the manuscript presented in an intelligible fashion and written in standard English?

PLOS ONE does not copyedit accepted manuscripts, so the language in submitted articles must be clear, correct, and unambiguous. Any typographical or grammatical errors should be corrected at revision, so please note any specific errors here.

Reviewer #2: Yes

Reviewer #4: Yes

Reviewer #5: Yes

Reviewer #6: Yes

**********

6. Review Comments to the Author

Please use the space provided to explain your answers to the questions above. You may also include additional comments for the author, including concerns about dual publication, research ethics, or publication ethics. (Please upload your review as an attachment if it exceeds 20,000 characters)

Reviewer #2: Thank you so much for addressing all comments properly. I understood your concern about your objectives.

Reviewer #4: While a statement has been provided in the submission system, it is not included within the main manuscript text. Please add a dedicated “Data Availability” section in the manuscript to comply with journal requirements.

Reviewer #5: The authors have adequately addressed the previous reviewers' comments and present a clear, scientific study of the impact of climate on dengue incidence in India.

Reviewer #6: Comments on the manuscript entitled “ Spatial Distribution and Risk Assessment of Dengue Incidence at District Level Across Major Climatic Zones in India”

The study provides a comprehensive analysis of the spatial and temporal distribution of dengue incidence in various climatic zones of India using surveillance data at the district level from the National Programme for 13 years (2010-2022). The analysis is based on relatively updated data and analytical methods aiming to assess the significance of variability between the major climatic zones in dengue incidence and its clustering. The results are relevant to the public health programme as the information may be useful for planning - targeting and prioritising areas – for preventive measures.

However, there are a few minor corrections and clarifications that need to addressed, which are mentioned below.

Table. 1. Column 4: Please explain how these mean values for Annual Incidence Rate, Peak Annual Incidence Rate, Mean Annual Cases and Peak Annual Mean Cases were calculated so that it will be clear to the general readers.

Line 217 – 222: “Aw also contributed to the largest proportion of the population in 2022 (32.57%) and mean annual cases (44,906.77), suggesting a strong relationship between population density in tropical savanna regions and disease transmission. Similarly, climate zones such as Temperate dry winter (Cwa) and hot arid (BSh) reported high incidence rates and substantial case counts, with Cwa covering the largest population proportion (36.91%) and reporting 22,188.54 mean annual cases”. – this part of the para does not read well. It is not clear if the mean annual cases or incidence rates were compared with the proportion population. For a 32.57% population, the number of cases were 44,906 whereas for 36.91% population, the number of cases was only 22,188.54. Needs clarity.

Table 2.: Post-hoc Comparison (pair-wise Wilcoxon test), Z1/Z3: The P values for the years 2011 & 2012 indicate that there is no significant difference. But, it is shown that these are significant; Table - Foot Note: Significant at P>0.05 – to be corrected as P<0.05.

Line, 236 -238: Though the AIR indicated a significant upward trend in these zones, there is a wide variability as indicated by 95% CI. This is to be mentioned and discussed.

The manuscript needs minor revision.

**********

7. PLOS authors have the option to publish the peer review history of their article (what does this mean?). If published, this will include your full peer review and any attached files.

If you choose “no”, your identity will remain anonymous but your review may still be made public.

**Do you want your identity to be public for this peer review?** For information about this choice, including consent withdrawal, please see our Privacy Policy.

Reviewer #2: No

Reviewer #4: No

Reviewer #5: No

Reviewer #6: No

**********

[NOTE: If reviewer comments were submitted as an attachment file, they will be attached to this email and accessible via the submission site. Please log into your account, locate the manuscript record, and check for the action link "View Attachments". If this link does not appear, there are no attachment files.]

To ensure your figures meet our technical requirements, please review our figure guidelines: https://journals.plos.org/plosone/s/figures

You may also use PLOS’s free figure tool, NAAS, to help you prepare publication quality figures: https://journals.plos.org/plosone/s/figures#loc-tools-for-figure-preparation.

NAAS will assess whether your figures meet our technical requirements by comparing each figure against our figure specifications.

Attachment Submitted filename: Comments_Spatio-temporal pattern_Dengue_13.04.2026.docx

# Author comment: Author response to Decision Letter 2

**DOI:** 10.1371/journal.pone.0350325.r004

27 Apr 2026

Authors Response to Reviewers

Revision no. 2

Manuscript Title: Spatial Distribution and Risk Assessment of Dengue Incidence at District Level Across Major Climatic Zones in India

Manuscript No. PONE-D-25-42541R2

Reviewer #2:

Thank you so much for addressing all comments properly. I understood your concern about your objectives.

We sincerely thank the reviewer for the positive feedback and for taking the time to carefully evaluate our manuscript. We are glad that the revisions adequately addressed the concerns raised.

Reviewer #4:

While a statement has been provided in the submission system, it is not included within the main manuscript text. Please add a dedicated “Data Availability” section in the manuscript to comply with journal requirements.

We thank the reviewer for this observation. As per the journal’s procedure, the data availability statement was previously provided in the submission system. The statement reads: "All relevant data are within the manuscript and its Supporting Information files," which will be typeset upon acceptance of the manuscript.

Reviewer #5:

The authors have adequately addressed the previous reviewers' comments and present a clear, scientific study of the impact of climate on dengue incidence in India.

We sincerely thank the reviewer for the positive and encouraging feedback, and for recognizing the scientific merit of our study. We are glad that the revisions met the reviewer's expectations and that the manuscript's objectives and findings are clearly presented.

Reviewer #6:

The study provides a comprehensive analysis of the spatial and temporal distribution of dengue incidence in various climatic zones of India using surveillance data at the district level from the National Programme for 13 years (2010-2022). The analysis is based on relatively updated data and analytical methods aiming to assess the significance of variability between the major climatic zones in dengue incidence and its clustering. The results are relevant to the public health programme as the information may be useful for planning - targeting and prioritising areas – for preventive measures.

We sincerely thank the reviewer for the thorough review of our manuscript. We are pleased that the reviewer recognized the comprehensiveness of our analysis, the relevance of the 13-year district-level surveillance data, and the public health significance of our findings.

However, there are a few minor corrections and clarifications that need to addressed, which are mentioned below.

1. Table. 1. Column 4: Please explain how these mean values for Annual Incidence Rate, Peak Annual Incidence Rate, Mean Annual Cases and Peak Annual Mean Cases were calculated so that it will be clear to the general readers.

We thank the reviewer for this helpful suggestion. We have added a footnote to Table 1 (Line 222-229) to clarify the calculation method for all four metrics, as described below:

Mean Annual Incidence Rate (per 100,000 people): Annual incidence rates were calculated for each district as ([cases ÷ projected population] × 100,000) and averaged across all 13 study years (2010-2022) and districts within each climate zone.

Peak Mean Annual Incidence Rate (per 100,000 people): The three highest annual incidence rates during 2010-2022 were identified for each climate zone and averaged. Mean Annual Cases: The total number of cases across all districts within each climate zone was summed for each year, and then averaged over the study years.

Peak Annual Mean Cases: The total number of cases across all districts within each climate zone was summed for each year, and then averaged across the three peak years.

Also, we have standardized all numerical values in Table 1 to two decimal places for consistency and uniformity across the table.

2. Line 217 – 222: “Aw also contributed to the largest proportion of the population in 2022 (32.57%) and mean annual cases (44,906.77), suggesting a strong relationship between population density in tropical savanna regions and disease transmission. Similarly, climate zones such as Temperate dry winter (Cwa) and hot arid (BSh) reported high incidence rates and substantial case counts, with Cwa covering the largest population proportion (36.91%) and reporting 22,188.54 mean annual cases”. – this part of the para does not read well. It is not clear if the mean annual cases or incidence rates were compared with the proportion population. For a 32.57% population, the number of cases were 44,906 whereas for 36.91% population, the number of cases was only 22,188.54. Needs clarity.

We thank the reviewer for this helpful observation. We have revised the paragraph for better clarity (line no. 201-207).

3. Table 2.: Post-hoc Comparison (pair-wise Wilcoxon test), Z1/Z3: The P values for the years 2011 & 2012 indicate that there is no significant difference. But, it is shown that these are significant; Table – Foot Note: Significant at P>0.05 – to be corrected as P<0.05.

We sincerely apologize for this typographical error in the table footnote. This has been corrected in the revised manuscript. We confirm that the p-values for the years 2011 and 2012 in the Z1/Z3 comparison do not reach statistical significance at P<0.05, and the significance markers for these years have been removed accordingly.

4. Line, 236 -238: Though the AIR indicated a significant upward trend in these zones, there is a wide variability as indicated by 95% CI. This is to be mentioned and discussed.

We thank the reviewer for this important observation. We agree that while the AIR indicated statistically significant upward trends in ET, Cfa, and Dfb, the wide 95% confidence intervals reflect considerable variability in these estimates. We have revised the text (line no. 247-255) to acknowledge this variability, attributing it to the small number of districts, low mean annual case counts, and negligible population proportions in these zones, which render the trend estimates sensitive to minor fluctuations in case numbers.

Attachment Submitted filename: Response to Reviewers.docx

# Peer review: Decision Letter 2

**DOI:** 10.1371/journal.pone.0350325.r005

**Authors:** Rajib Chowdhury

13 May 2026

Spatial Distribution and Risk Assessment of Dengue Incidence at District Level Across Major Climatic Zones in India

PONE-D-25-42541R2

Dear Dr. Behera,

We’re pleased to inform you that your manuscript has been judged scientifically suitable for publication and will be formally accepted for publication once it meets all outstanding technical requirements.

Within one week, you’ll receive an e-mail detailing the required amendments. When these have been addressed, you’ll receive a formal acceptance letter and your manuscript will be scheduled for publication.

An invoice will be generated when your article is formally accepted. Please note, if your institution has a publishing partnership with PLOS and your article meets the relevant criteria, all or part of your publication costs will be covered. Please make sure your user information is up-to-date by logging into Editorial Manager at Editorial Manager® and clicking the ‘Update My Information' link at the top of the page. For questions related to billing, please contact billing support.

If your institution or institutions have a press office, please notify them about your upcoming paper to help maximize its impact. If they’ll be preparing press materials, please inform our press team as soon as possible -- no later than 48 hours after receiving the formal acceptance. Your manuscript will remain under strict press embargo until 2 pm Eastern Time on the date of publication. For more information, please contact onepress@plos.org.

Kind regards,

Rajib Chowdhury, M.Sc.; MPH

Academic Editor

PLOS One

Additional Editor Comments (optional):

Reviewers' comments:

Reviewer's Responses to Questions

**Comments to the Author**

1. If the authors have adequately addressed your comments raised in a previous round of review and you feel that this manuscript is now acceptable for publication, you may indicate that here to bypass the “Comments to the Author” section, enter your conflict of interest statement in the “Confidential to Editor” section, and submit your "Accept" recommendation.

Reviewer #2: All comments have been addressed

Reviewer #4: All comments have been addressed

Reviewer #5: All comments have been addressed

**********

2. Is the manuscript technically sound, and do the data support the conclusions?

The manuscript must describe a technically sound piece of scientific research with data that supports the conclusions. Experiments must have been conducted rigorously, with appropriate controls, replication, and sample sizes. The conclusions must be drawn appropriately based on the data presented.

Reviewer #2: Yes

Reviewer #4: Yes

Reviewer #5: Yes

**********

3. Has the statistical analysis been performed appropriately and rigorously?

Reviewer #2: Yes

Reviewer #4: Yes

Reviewer #5: Yes

**********

4. Have the authors made all data underlying the findings in their manuscript fully available?

The PLOS Data policy requires authors to make all data underlying the findings described in their manuscript fully available without restriction, with rare exception (please refer to the Data Availability Statement in the manuscript PDF file). The data should be provided as part of the manuscript or its supporting information, or deposited to a public repository. For example, in addition to summary statistics, the data points behind means, medians and variance measures should be available. If there are restrictions on publicly sharing data—e.g. participant privacy or use of data from a third party—those must be specified.

Reviewer #2: Yes

Reviewer #4: Yes

Reviewer #5: Yes

**********

5. Is the manuscript presented in an intelligible fashion and written in standard English?

PLOS ONE does not copyedit accepted manuscripts, so the language in submitted articles must be clear, correct, and unambiguous. Any typographical or grammatical errors should be corrected at revision, so please note any specific errors here.

Reviewer #2: Yes

Reviewer #4: Yes

Reviewer #5: Yes

**********

6. Review Comments to the Author

Please use the space provided to explain your answers to the questions above. You may also include additional comments for the author, including concerns about dual publication, research ethics, or publication ethics. (Please upload your review as an attachment if it exceeds 20,000 characters)

Reviewer #2: Dear Author,

Thanks for addressing all comments. It should be published due to recent emerging issue.

Thanks,

Reviewer #4: All the comments have been satisfactorily addressed by the authors in the revised manuscript in detail.

Reviewer #5: The authors have appropriately corrected the error in Table 2 as highlighted in the last review and addressed all other comments to clarify the text. I do not have any reservations in accepting this study for publication.

**********

7. PLOS authors have the option to publish the peer review history of their article (what does this mean?). If published, this will include your full peer review and any attached files.

If you choose “no”, your identity will remain anonymous but your review may still be made public.

**Do you want your identity to be public for this peer review?** For information about this choice, including consent withdrawal, please see our Privacy Policy.

Reviewer #2: No

Reviewer #4: No

Reviewer #5: No

**********

# Editor report: Acceptance letter

**DOI:** 10.1371/journal.pone.0350325.r006

**Authors:** Rajib Chowdhury

PONE-D-25-42541R2

PLOS One

Dear Dr. Behera,

I'm pleased to inform you that your manuscript has been deemed suitable for publication in PLOS One. Congratulations! Your manuscript is now being handed over to our production team.

At this stage, our production department will prepare your paper for publication. This includes ensuring the following:

* All references, tables, and figures are properly cited

* All relevant supporting information is included in the manuscript submission,

* There are no issues that prevent the paper from being properly typeset

You will receive further instructions from the production team, including instructions on how to review your proof when it is ready. Please keep in mind that we are working through a large volume of accepted articles, so please give us a few days to review your paper and let you know the next and final steps.

Lastly, if your institution or institutions have a press office, please let them know about your upcoming paper now to help maximize its impact. If they'll be preparing press materials, please inform our press team within the next 48 hours. Your manuscript will remain under strict press embargo until 2 pm Eastern Time on the date of publication. For more information, please contact onepress@plos.org.

You will receive an invoice from PLOS for your publication fee after your manuscript has reached the completed accept phase. If you receive an email requesting payment before acceptance or for any other service, this may be a phishing scheme. Learn how to identify phishing emails and protect your accounts at https://explore.plos.org/phishing.

If we can help with anything else, please email us at customercare@plos.org.

Thank you for submitting your work to PLOS ONE and supporting open access.

Kind regards,

PLOS ONE Editorial Office Staff

on behalf of

Dr. Rajib Chowdhury

Academic Editor

PLOS One
